# Molecular characterization of a tetra segmented ssDNA virus infecting *Botrytis cinerea* worldwide

**DOI:** 10.1186/s12985-023-02256-z

**Published:** 2023-12-19

**Authors:** Ana Ruiz-Padilla, Massimo Turina, María A. Ayllón

**Affiliations:** 1 Centro de Biotecnología y Genómica de Plantas, Universidad Politécnica de Madrid-Instituto Nacional de Investigación y Tecnología Agraria y Alimentaria (UPM-INIA/CSIC), Pozuelo de Alarcón, Madrid, Spain; 2 Institute for Sustainable Plant Protection, National Research Council of Italy, Torino, Italy; 3 Departamento de Biotecnología-Biología Vegetal, Escuela Técnica Superior de Ingeniería Agronómica, Alimentaria y de Biosistemas, Universidad Politécnica de Madrid (UPM), Madrid, Spain

**Keywords:** Hypovirulence, Fungi, ssDNA viruses, Multisegmented genome, Replication

## Abstract

**Background:**

Family *Genomoviridae* was recently established, and only a few mycoviruses have been described and characterized, and almost all of them (Sclerotinia sclerotiorum hypovirulence-associated DNA virus 1, Fusarium graminearum gemyptripvirus 1 and Botrytis cinerea gemydayirivirus 1) induced hypovirulence in their host. Botrytis cinerea ssDNA virus 1 (BcssDV1), a tetrasegmented single-stranded DNA virus infecting the fungus *Botrytis cinerea*, has been molecularly characterized in this work.

**Methods:**

BcssDV1 was detected in Spanish and Italian *B. cinerea* field isolates obtained from grapevine. BcssDV1 variants genomes were molecularly characterized via NGS and Sanger sequencing. Nucleotide and amino acid sequences were used for diversity and phylogenetic analysis. Prediction of protein tertiary structures and putative associated functions were performed by AlphaFold2 and DALI.

**Results:**

BcssDV1 is a tetrasegmented single-stranded DNA virus. The mycovirus was composed by four genomic segments of approximately 1.7 Kb each, which are DNA-A, DNA-B, and DNA-C and DNA-D, that coded, respectively, for the rolling-circle replication initiation protein (Rep), capsid protein (CP) and two hypothetical proteins. BcssDV1 was present in several Italian and Spanish regions with high incidence and low variability among the different viral variants. DNA-A and DNA-D were found to be the more conserved genomic segments among variants, while DNA-B and DNA-C segments were shown to be the most variable ones. Tertiary structures of the proteins encoded by each segment suggested specific functions associated with each of them.

**Conclusions:**

This study presented the first complete sequencing and characterization of a tetrasegmented ssDNA mycovirus, its incidence in Spain and Italy, its presence in other countries and its high conservation among regions.

**Supplementary Information:**

The online version contains supplementary material available at 10.1186/s12985-023-02256-z.

## Background

Family *Genomoviridae* was accepted in 2016 by the ICTV [1] as the first one including single-stranded DNA (ssDNA) viruses infecting fungi and insects. These viruses are classified in the phylum *Cressdnaviricota* and the recently defined order *Geplafuvirales*. They possess a circular ssDNA genome of 1.8–2.4 kb with two open reading frames (ORF) encoding a rolling-circle replication initiation protein (Rep) and a capsid protein (CP). Since its establishment, more than 1450 viral genomes (viral database NCBI) have been described inside the family. Genomoviruses have been classified in ten genus: *Gemycircularvirus*, *Gemyduguivirus*, *Gemygorvirus*, *Gemykibivirus*, *Gemykolovirus*, *Gemykrogvirus*, *Gemykroznavirus*, *Gemytondvirus*, *Gemyvongvirus* and *Gemyptripvirus* [2].

Sclerotinia sclerotiorum hypovirulence-associated DNA virus 1 (SsHADV-1) was the first ssDNA virus demonstrated to infect fungi [3]. SsHADV-1 has an ambisense circular genome of 2.2 kb with two ORFs, one in positive sense encoding a CP and the other in negative sense coding for the Rep. It also contains two intergenic regions, one of them including the origin of replication (Ori) TAATATTAT [4]. SsHADV1 is encapsidated forming isometric viral particles that were able to infect *Sclerotinia sclerotiorum* extracellularly and reduce fungal lesions in *Brassica napus*. SsHADV1 was classified in the genus *Gemycircularvirus*. Since its discovery, only two more new mycoviruses of the family *Genomoviridae* have been described. Fusarium graminearum gemyptripvirus 1 (FgGMTV1) was the first described tripartite genomovirus composed by DNA-A, DNA-B and DNA-C coding for Rep, CP, and a hypothetical protein, respectively [5]. FgGMTV1 has been classified within the new genus *Gemytripvirus* (gemini-like myco-infecting tripartite virus) [6]. Multipartitism has been hypothetically explained because of the formation of defective genomes which can be posteriorly, transreplicated and/or transencapsidated, generating new components in the genome [7], which could be beneficial for viral evolution.

Botrytis cinerea ssDNA virus 1 (BcssDV1) [8], Botrytis cinerea genomovirus 1 (BcGV1) [9] and Botrytis cinerea gemydayirivirus 1 (BcGDV1) [10] have been described infecting the necrotrophic fungus *Botrytis cinerea*. They all had a monosegmented genome of approximately 1.7 Kb with at least one ORF encoding for the Rep that contained all conserved motifs of replication-associated proteins of this type of viruses (motifs I to III, GRS domain, Walker A and B, and motif C) [11]. BcssDV1 was first identified infecting *B. cinerea* field isolates from Spanish and Italian vineyards. It contained an ORF that coded for a 380 amino acid (aa) protein containing Rep conserved domains. AACAGTAC was proposed as the nonanucleotide of the Ori. BcGV1 was detected in *B. cinerea* strains isolated from bean leaves in China. Its genome contained two ORFs: ORF1 coding for a 321 aa rep and ORF2 coding for a 129 aa hypothetical protein. These two ORFs were separated by two intergenic regions, a large intergenic region (LIR) and a small intergenic region (SIR), and inside LIR sequence, the nonanucleotide TAACAGTAC was marked as possible initiation site for viral DNA replication. BcGDV1 was identified in fungal isolates obtained from asymptomatic plants in New Zealand. It was composed by three ORFs: ORF1 was coding for a 321 aa protein, while ORF2 and ORF3 were overlapping, and encoded for hypothetical proteins of 124 and 97 aa, respectively. ORF1 and overlapping ORF2 and 3 were separated by LIR and SIR sequences, and CTATCAACAC was identified as the putative nonanucleotide sequence in the loop of the stem-loop structure in the Ori region. Reps sequences of BcssDV1, BcGV1 and BcGDV1 shared an identity of 98% among them, so according to demarcation criteria that no sequences from different species share > 78% aa identity in the Rep protein [6], they should be considered as members of the same viral species.

As well as SsHADV1, both FgGV1 and BcGDV1 showed hypovirulence traits in its respective hosts *F. graminearum* and *B. cinerea* [10, 12]. FgGV1 infectious clones inoculated in protoplasts were proven to induce hypovirulence in *F. graminearum* [5] and BcGDV2 purified particles applied to the growing margins of a *B. cinerea* virus-free strain resulted in a hypovirulent phenotype of the new infected strain in canola leaves. Additionally, BGDaV1 methylation studies proved that *B. cinerea* host defense machinery targets the BGDaV1 genome down-regulating BGDaV1 RNA accumulation. This silencing results in a long-term mutualistic infection relation that makes both survive during viral infection [10].

*B. cinerea* associated genomoviruses are potential biotechnological tools to understand virus-fungi-plant interactions and to provide solutions for the control of gray mould diseases. In fact, *B. cinerea* is one of the most important and aggressive plant-pathogenic fungus worldwide [13]. It infects a wide range of crops, in the field and in postharvest, in form of mycelia, spores or sclerotia for long periods of time. Chemical fungicides, as the main control method, tends to change to more sustainable solutions as mycovirus-derived control methods [14].

In this work, we show the molecular characterization of several variants of BcssDV1 identified in different isolates of Italy and Spain. It is demonstrated that BcssDV1 genome is composed by four segments, instead of a single one as was previously published [8]. Additionally, in silico alignment and prediction methods helped us to elucidate the putative functions of the hypothetical proteins in the interactions with their host.

## Methods

### Fungal strains

*B. cinerea* field isolates were originally obtained from infected grapevines (*Vitis vinifera*) of different regions in Italy and Spain [8]. *B. cinerea* B05.10 strain, free of mycoviruses, was used as a control. All strains were stored at -80 °C in glycerol and refreshed in PDA plates at 24 °C for 7 days.

### Bioinformatic analysis of NGS data

RNAs from *B. cinerea* mycelia were extracted as described previously [8]. Briefly, 100 mg of dry mycelia were lysed and subjected to total RNA extraction with Spectrum Plant Total RNA kit. RNA concentration was measured with Nanodrop and quality was tested by an electrophoresis 1% agarose gel. Total RNAs were analyzed by high-throughput sequencing as described previously to determine the mycovirome [8]. Briefly, reads were processed and assembled in transcripts using Trinity. Contigs were then compared against a viral database using Blastx and mycoviruses were identified. After the molecular characterization of the BcssDV1 genome, its nucleotide (nt) and aa sequences were used to create a reference database. In Ubuntu desktop, a local Blast was run using Translated Query-Protein Subject BLAST 2.9.0+ and the parameter e-value set as 0,01 to find other assembled contigs that contain regions similar or highly similar. These regions are denominated common regions, as previously described for FgGV1 [5]. Resulted contigs were analyzed to determine whether there were or not additional segments of BcsDV1 genome. Genome and alignments visualization were performed with Geneious® software. Secondary structures of DNA replication origin were predicted with UNAfold [15].

### Viral particle enrichment and viral DNA extraction

Mycelia of *B. cinerea* strain IBC1, grown at 24 °C for 10 days on PDB, were harvested and frozen in N_2_ liquid. Viral particles were purified following a previous modified protocol [9]. Briefly, 30 g of mycelia were grinded to a fine powder that was suspended in an extraction buffer and then centrifuged at 18,000xg for 20 min to remove large particles corresponding to mycelial debris. The resultant supernatant was ultracentrifuged in a 2 ml 20% sucrose cushion for 2 h at 27,000 × g at 4 °C to precipitate all particles. The obtained pellet was resuspended in 1 ml of 0.1 M sodium phosphate buffer and then centrifuged at 16,000 × g for 20 min to clarify the suspensions and remove cellular debris and other contaminants.

Viral particles were treated with nucleases S1 (NEB) and RQ1 (Promega) to remove any non-encapsidated viral DNA remaining in the sample. Viral particle solution was used as template for PCR amplification to detect contaminant viral DNA that could have been released out of the viral particles during treatment. Resulting suspensions treated with nucleases were used as negative control of the PCR. Viral particles with no external DNA contamination were used to extract the viral DNA. Viral particles were resuspended in a total volume of 100 µL of SM buffer (0.1 M NaCl, 50 mM Tris–HCl (pH 7.4), 10 mM MgSO4) and viral DNA was extracted using the High Pure Viral Nucleic Acid Kit (Roche), following manufacturer’s instructions.

Presence of viral DNA was confirmed by amplifying specific regions of BcssDV1 genomic segments DNA-A and DNA-B with primers rep4_Fw and rep Rev, and 327_4Fw and 3274_Rev, respectively (Table 1).

**Table 1:** List of primers used for characterization and detection of genomes of BcssDV1

Primer	Segment	Sequence (5’–3’)
Rep_Fw4	DNA-A	CCATTACGATGGCAGTCCACAC
Rep_Fw3	DNA-A	AGGCTCGTTTAGGCATATTCC
Rep_Rev	DNA-A	CAC GTG ACG ATT TGC CTA GAG ATC
Rep_Rev5	DNA-A	CAGCCAATACCGTCGACGAATT
Rep_Fw	DNA-A	CTC TTA TAG TCA GAG CTC CAC AC
Rep_rev3	DNA-A	GGAAGTGGTGCTTATTGTGAATAC
327_Fw	DNA-B	CTAGCTCCGCTATATCCTGCTCG
327_Rev5	DNA-B	ATATTTGTTGGCAATTTGGGCG
327_Fw4	DNA-B	GCCCTGTTGGCTTAGATATGC
327_Rev3	DNA-B	GTGATAGCCTGTAAGGCGCTGTAA
225_Fw3	DNA-C	GCGACCATAAATCTAGCGAACACG
225_Rev6	DNA-C	CTCAGGCTCCGCGGTAACAACC
270_Fw5	DNA-D	TATCCTCAGGATAGCCTATAAATAC
270_Rev3	DNA-D	TCGCTAAACCTGAATAACAGTCC
270_Fw3	DNA-D	CTGGTATAAAGCCCAAGCAAATG
270_Rev4	DNA-D	TTCCAGTGGGAATTTGTCTGC
pJet1.2_Fw	pJet1.2	CGACTCACTATAGGGAGAGCGGC
pJet1.2_Rev	pJet1.2	AAGAACATCGATTTTCCATGGCAG
FG_det_Fw	DNA-A	CGTGTCACCAGAGATCGTATG
FG_det_Rev	DNA-A	GTGATTCACCTACATACGGCTC
rep_1505R	DNA-A	AGAAACCCCTGGAAGCATCG
DNAA_846_Rev	DNA-A	CACGCCTACGTCGAAGCACCAACAC
Rep_Fw4	DNA-A	CCATTACGATGGCAGTCCACAC
327_Rev	DNA-B	CCTCCTTCATGTTCGTCACACC
270_Fw	DNA-D	GCT TTA TTA CGA CGT GAT ACG ATG
270_Rev	DNA-D	GAT GCT GAT GAA AAT TCC AGT GGG
BcssDV1_DNAC_ORF_Fw	DNA-C	ATGTCCGATTCAGAGTTCGATGTTTC
BcssDV1_DNAC_ORF_Rev	DNA-C	TCATGTTGCGAACTCATACAACCTGG
BcssDV1_DNAD_ORF_Fw	DNA-D	ATGCAGACAAATTCCCACTGGAATTTTC
BcssDV1_DNAD_ORF_Rev	DNA-D	TTAATTTCTCCCACCTAGACTACGC

Names, target of amplification and sequence of primers used for characterization and detection of genomes of BcssDV1 in pools of field isolates from Spain and Italy

All amplifications were performed using CloneAmp polymerase (Takara), applying a denaturalization step of 98 °C for 30 s, and 35 cycles of denaturation at 98 °C 10 s, annealing at 55–57 °C for 15 s and elongation at 72 °C for 30 s/Kb, and a final extension cycle of 72 °C for 7 min.

### Determination of the complete sequence of BcssDV1

Viral DNA, extracted from viral particles purified from the mycelia of the field isolate IBC1, was used as template to amplify full genomes using specific primers for each segment (designed in adjacent positions based on the sequence obtained by NGS). Primers Rep_Fw4 and Rep_Rev5 were used to amplify the full-length sequence of segment DNA-A, 327_Fw and 327_Rev5 for segment DNA-B, 225_Fw3 and 225_Rev6 were used for DNA-C and 270_Fw5 and 270_Rev3 for segment DNA-D (Table 1). To confirm the independence and circularity of each of the four genomic segments, primers were designed to amplified and sequence overlapping regions. For DNA-A, the set of primers used were rep3_Rev and rep_Fw and rep_Rev; for DNA-B, 327_3_Fw, 327_3_Rev, 327_Rev; for DNA-C, 225_Fw, 225_Rev and 225_Fw3 and, for DNA-D, 270_Fw3, 270_Rev3, 270_Fw and 270_Rev (Table 1). Additionally, approximately 20 ng of DNA were used for rolling circle amplification (RCA) using TempliPhi (GE Healthcare), following manufacter’s instructions, to specifically amplify the circular genome of BcssDV1. Digestion of RCA products were performed with CutSmart buffer for SpHI, SacII and AclI restriction enzymes, and with Buffer 2.1 for HindIII (New England Biolabs). Approximately, 6 µl of RCA products were incubated for 3 h at 37 °C in a total volume of 25 µl with a total concentration of 1 × Buffer and 3 units of enzyme.

Full genome of each of the four segments from BCI1 variant, were independently cloned in the vector pJET1.2 by blunting ligation using CloneJet PCR Cloning kit (Thermofisher). Positive clones were sequenced using pJET1.2 Fw and Rev primers and additional internal primers to complete the genomic sequence by paired sequences alignment.

### Sequencing of BcssDV1 variants

Sequences of all segments of other variants of BcssDV1 infecting other pools were obtained as explained in Sect. "Bioinformatic analysis of NGS data" and by assembling original reads using as reference previously identified segments. For incomplete in silico sequences of BcssDV1 variants, PCR amplification from total cDNA obtained from the total RNA of some individual field isolates was performed to obtain full-genome sequences. Complete sequences of DNA-A were obtained by Sanger sequencing using primers rep4_Fw, rep5_Rev, rep1_Reb, FG_det_Fw, FG_det_Rev, rep_1505R and DNAA_846_Rev (Table 1). Similarly, DNA-D complete sequences were obtained using 270_Fw3, 270_Rev3 and 270_Fw5 (Table 1). Full- genome sequences of each segment were obtained by assembling all Sanger sequences of each segment of BcssDV1 using Geneious®.

For phylogenetic analyses, nt sequences of variants of each segment were aligned using Clustal Omega and trimmed manually by Geneious to obtain equal length for all of them. ORFs of resulted trimmed sequences were translated and resulting proteins were aligned using Clustal Omega. Sequences sharing 100% of identity inside same pool were discarded for phylogenetic analysis.

### Phylogenetic and protein structure analysis

MEGA X [16] was used to estimate the overall mean distance in the entire population (total number of sequences of all regions). For phylogenetic reconstruction, nt and aa sequence alignments were exported to IQ-TREE server (http://iqtree.cibiv.univie.ac.at/) and subjected to a best DNA/protein model analysis [17]. Phylogenetic trees for each segment were constructed using the maximum likehood (ML) method, applying the best model obtained for each one and a test with a bootstrap of 1000 replicates. Phylogenetic trees of nt sequences were inferred by ML using next models of substitution, DNA-A, TNe + G4 + F; DNA-B, TN + G4 + F; DNA-C, TNe + G4; DNA-D, TN + F + G4. Phylogenetic trees of aa sequences were inferred by ML using next models of substitution, DNA-A, VT + I + G4 + F; DNA-B, VT + F; DNA-C, JTT + G4; DNA-D, FLU + G4. Trees were visualized and edited using software MEGA X.

The number of base substitutions per site from mean diversity calculations for the entire population were calculated using MEGAX [18]. Their associated standard error estimate(s) were obtained by a bootstrap procedure (1000 replicates). Analyses were conducted using the Tamura-Nei model [19]. The rate variation among sites was modelled with a gamma distribution (shape parameter -1). In addition, pairwise identities were also calculated at nt and aa level using Clustal Omega. Tertiary structure of BcssDV1 isolate IBC1 encoded proteins were modelled with AlphaFold2, a computational approach capable of predicting protein structures with high accuracy and based on neural network [20]. Quality of the predicted structures was analyzed by QMEAN (https://swissmodel.expasy.org/qmean/) [21]. Predicted protein structures of DNA-A and DNA-B were compared with Rep and CP of SsHADV1 and FgGV1. Predicted structures of DNA-C and DNA-D of BcssDV1 were also compared with hypothetical protein coding in DNA-C of FgGV1. Additionally, prediction of the tridimensional structure of all BcssDV1 proteins was also performed with Phyre2 server [22] and DALI [23]. In Phyre2, aa sequence of each segment was introduced in the server to find structural homologies using a normal model or an intensive and sensitive one. In DALI (Distance matrix alignment), tertiary structures of proteins of DNA-A, DNA-B, DNA-C and DNA-D, previously predicted by AlphaFold2, were submitted to perform structural comparison.

### Detection of BcssDV1 in *B. cinerea* field isolates

Total RNAs of individual field isolates included in each pool where BcssDV1 was found were tested to validate the presence of BcssDV1. Detection was carried out by one-step RT-PCRs using specific primers for DNA-A: rep_Fw and rep Rev3; for DNA-B: 327_Fw4 and 327_Rev3; for DNA-C: BcssDV1_DNAC_ORF_Fw and BcssDV1_DNAC_ORF_Rev; and for DNA-D: BcssDV1_DNAD_ORF_Fw and BcssDV1_DNAD_ORF_Rev (Table 1). PCR products were visualized in 2% agarose gel.

## Results

### Analysis of the multisegmented nature of BcssDV1 genome

*B. cinerea* field isolates were previously obtained from infected grapes of vineyards of Italy and Spain and their mycovirome was determined [8]. A new ssDNA virus (BcssDV1, Genbank accession no. MN625247) was discovered and characterized. The sequence previously characterized of BcssDV1 (from now named DNA-A) was 1692 nt long and contained one ORF that coded for a 321 aa protein or a spliced-380 aa protein [8].

BcssDV1 was detected in transcripts of different pools of Italy and Spain, BCI1, BCI2, BCS8, BCS10, BCS11, BCS12, BCS13, BCS14, BCS15 and BCS17 ([8]; Table 2). Some transcripts had non-complete genomic sequence or were artifacts added by the assembler program used, but all of them contained the Rep conserved regions.

**Table 2:** List of pools, corresponding region, locations and number (#) of reads of DNA-A of BcssDV1

Pool	Region	Location (# isolates)	# of reads
BCI1	Lombardia	Lonato del Garda [10]	171,699
BCI2	Lombardia	Lonato del Garda [3], Moniga del Garda [5], Muscoline [1]	253
BCS8	Ribera del Duero	La Horra [9]	95,687
BCS10	Penedes	Villafranca del Penedés [1] Pla del Penedés [6]	49,896
BCS11	Penedes	Recaredo [9]	26,115
BCS12	Penedes	Recaredo [7]	86,738
BCS13	La Rioja	Matahón—FuenMayor [3], Andaverde—Labastida [1]	862
BCS14	La Rioja	Romeral—FuenMayor [12]	694
BCS15	La Rioja	Carretera—Baños del Ebro [10]	20,320
BCS17	La Rioja	Salmuera—San Vicente de la Sonsierra [8]	28,996

List of pools, region, locations of collection of corresponding *B. cinerea* isolates and number of reads mapping with BcssDV1

**Fig. 1:**
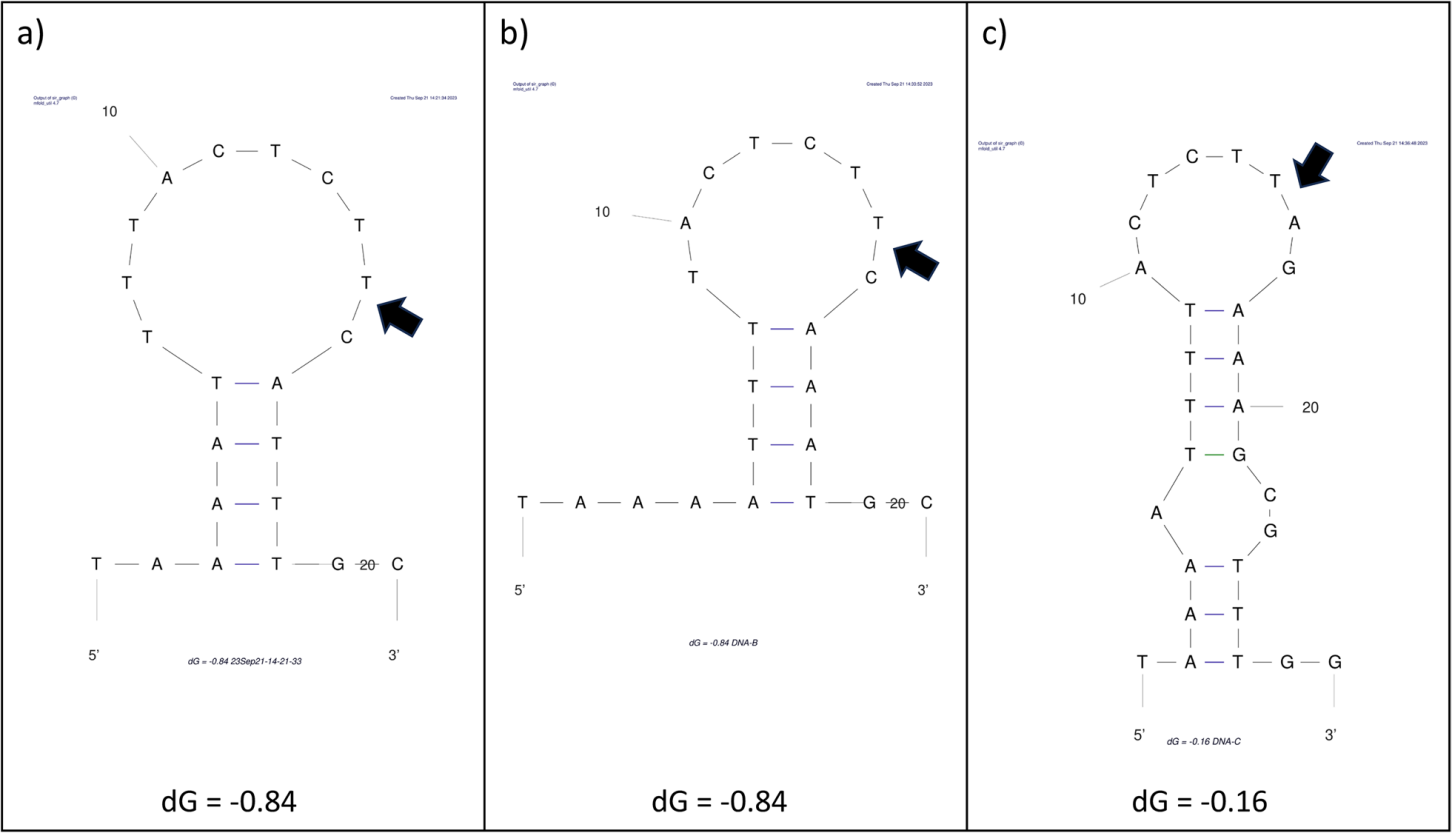
Stem-lop secondary structures. Diagrams showing stem-loop secondary structures of the sequence around the conserved nonanucleotide of the Ori in DNA-A and D (**a**), DNA-B (**b**) and DNA-C (**c**)

BcssDV1 was distributed in three regions of Spain (Ribera del Duero, Penedés and Rioja) and one single region of Italy (Lombardia). Reads were also mapped with SAMtools [24] and are presented in Table 2, showing quantities from 252 reads, in pool IBC2, to 171,699, in pool IBC1. In our previous study, beside BcssDV1 DNA-A, we found several other transcripts in different pools with the non-coding regions similar to the same regions in BcssDV1 DNA-A, suggesting that its genome could be composed by more than one segment, and further analyses started to determine the full sequence of all the putative segments of BcssDV1 genome. Transcripts were blasted against BcssDV1 genome to find additional genomic segments. After analyzing all resulting transcripts, two common regions, L-CR (Long Common Region) and S-CR (short common region) of approximately 260 and 38 nts, respectively, were found in three new putative genomic segments. All segments were aligned using Clustal Omega and two different putative nonanucleotide sequences were detected in S-CR of all segments. For DNA-A, DNA-B and DNA-D, nonanucleotide sequence was TACTCTT^CA and for DNA-C, TACTCTT^AG. The secondary structure of the region around the nonanucleotide sequence was also conserved among typical Ori sequences of viruses of the family *Genomoviridae* [1], showing the putative Ori of all BcssDV1 genomic segments in a loop of a stem-loop structure (Fig. 1).

### Molecular characterization of the complete multisegmented genome of BcssDV1

**Fig. 2:**
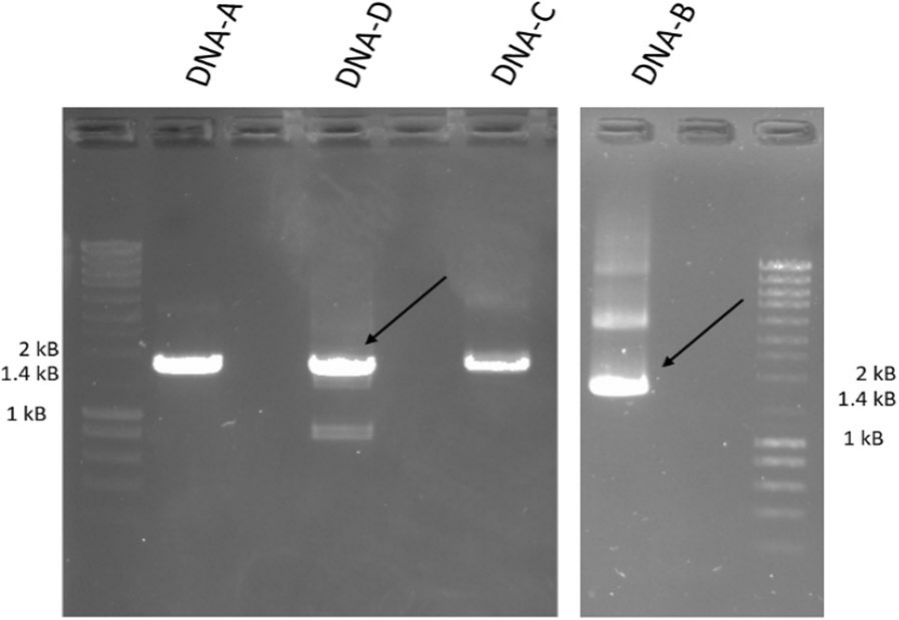
Amplification of full segments of BcssDV1 from viral DNA

Viral particles of BcssDV1 were purified from mycelia of the *B. cinerea* BCI1 strain, included in the IBC1 pool [8]. Viral DNA was successfully extracted from viral particles and used as template for the amplification of all four genomic segments. Full-genome segments were successfully amplified (Fig. 2) and cloned into pJET1.2 vector to determine their nt sequences by Sanger sequencing.

**Fig. 3:**
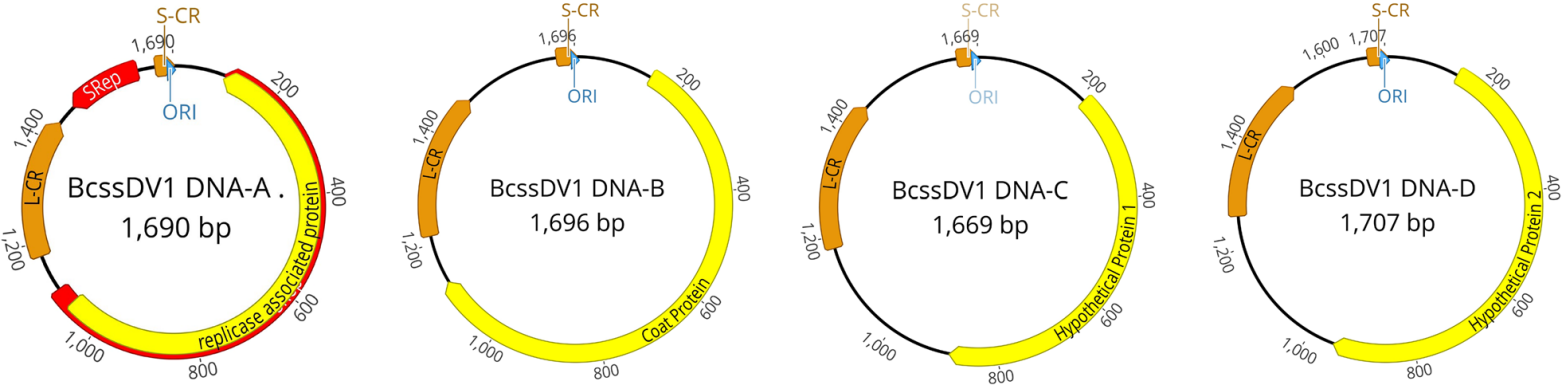
Schematic diagram of the genome organization of Botrytis cinerea ssDNA virus 1 (BcssDV1). Yellow arrows indicate the coding regions in the translation orientation, blue arrows indicate the origin of replication and orange arrows indicate L-CR and S-CR regions. ORI is marked as blue. The DNA-A protein of 380 aa, obtained by deletion of a putative intron, is marked in red

DNA-A segment of IBC1 was 1690 nts long, with a GC content of 49,1%, and contained one ORF in negative sense that encoded one protein of 321 aa (35,63 kDa), with conserved geminiviruses Rep motives. Deletion of a putative intron generates a new ORF coding for a spliced protein of 380 aa. The sequence of 321 aa shares 94,6% of identity at nt level and 98.1% at aa level with the previously described BCS11 variant (MN625247, this sequence was annotated in the opposite orientation, since the ORF coding for Rep is in negative sense the sequence has been corrected in the GenBank). BCS11 variant MN625247 was only present in two pools, while the new described variant of DNA-A IBC1 was detected in all analyzed pools. DNA-B segment was 1690 nts long, with a GC content of 48.5%, and contained one ORF in positive sense that encoded the putative mycoviral CP of 327 aa (36,3 kDa). DNA-C was 1669 nts long, with a GC content of 46.7%, and contained one ORF in positive sense that encoded a hypothetical protein of 225 aa (24,98 kDa), whose function is unknown. DNA-D was 1707 nts long, with a GC content of 45.8%, and contain one ORF that encoded one protein of 270 aa (29.97 kDa), also with unidentified function (Fig. 3). No conserved motifs were found in the aa sequences of DNA-B, DNA-C and DNA-D.

The results of the Blast search showed that segments DNA-A, DNA-B, DNA-C and DNA-D of IBC1 shared an identity of 91.5%, 97.5%, 91.4% and 93.9% at nt level, with segments DNA-A, DNA-B, DNA-C and DNA-D of Botrytis cinerea hypovirulence-associated DNA virus 1, respectively (GenBank Accession No. MT425546, unpublished). At amino acidic level, identities were 95.7%, 99.1%, 92.4% and 93.7%, respectively. These results indicated that both sequences are variants of the same mycovirus species. Rep of DNA-A sequence was also aligned with Reps of SsHADV1 and FgGV1 and shared an identity of 33.2% and 41%, respectively. However, same analysis with the associated CPs of the three mycoviruses resulted in lower non-significant identities of 12.2% and 13.2% between CP of DNA B and CPs of FgGV1 and SsHADV1, respectively. At nt level DNA-A and DNA-B of BcssDV1 shared an identity of 44.4% and 38.3% with DNA-A and DNA-B of FgGV1, respectively. For DNA-C and DNA-D of BcssDV1, the comparison was done with DNA-C of FgGV1, and they shared and identity of 25.7% and 26.8%, respectively. For the same species of *B. cinerea* ssDNA viruses, identities were higher than 85.7% for L-CR and 100% for S-CR. In comparison with FgGV1 genomic segments, identity was lower and variable between 30.4 and 48.8% for L-CR and 38.2–65.9% for S-CR.

BcssDV1 variants of pools BCI2, BCS8, BCS10, BCS11, BCS12, BCS13, BCS14, BCS15 and BCS17 were obtained. Table 3 shows GenBank accession number and the source of each nt sequence segment in each pool. Most contigs were obtained by Trinity assembling all NGS processed reads. Nevertheless, remaining contigs that could not be obtained with this method were assembled using Geneious assembler and BcssDV1- BCI1 genomic segments as reference. Due to the low number of reads of some segments, it was required to synthesize cDNA from original RNA extractions of isolates IBC18, Bc85, Bc91 and Bc114, included in pools BCI2, BCS13, BCS14 and BCS15, respectively. Full length segments were amplified and Sanger sequenced. However, DNA-B sequence obtained from BCI2 pool was partial and its complete sequence could not be determined. ORFs found in DNA-D segments were translated and resulted in aa sequences of the expected length of 270 aa (BC1, BCS10, BCS13, BCS14, BCS15, BCS17), but four sequences where shorter (243 aa for BCS8, BCS11 and BCS12).

**Table 3:** Genbank accession numbers and description of protocol followed for obtaining complete sequences of BcssDV1 segments

Pool	DNA-A	DNA-B	DNA-C	DNA-D
BCI1	C and S (OR146520) N and T (OR146507 and OR146508)	C and S (OR146542) N and T (OR146523, OR146524 and OR146525)	C and S (OR146564) N and T (OR146543, OR146544, OR146545, OR146546 and OR146547)	C and S (OR146577) N and G (OR146568)
CI2	RP and S (isolate IBC18) (OR146521)	* N and T*	N and T (OR146549)	N and G (OR146569)
BCS8	N and G (OR146515)	N and G (OR146539)	N and T (OR146550)	N and G (OR146570)
BCS10	N and T (OR146509 and OR146510)	N and T (OR146527 and OR146529)	N and T (OR146551 and OR146552)	N and G (OR158040)
BCS11	N and T (OR146511 and OR146512)	N and T (OR146531)	N and T (OR146553)	N and G (OR146572)
BCS12	N and T (OR146513) N and G (OR146516)	N and T (OR146532) N and G (OR146540)	N and T (OR146554, OR146555, OR146556, OR146557 and OR146558)	N and G (OR146573) N and T (OR146565)
BCS13	N and G (OR146517)	N and T (OR146534) N and G (OR146541)	N and T (OR146559)	RP and S (isolate Bc85) (OR146575)
BCS14	N and G (OR146518)	N and T (OR146535)	N and T (OR146560)	RP and S (isolate Bc91) (OR146576)
BCS15	N and G (OR146519) RP and S (OR146522)	N and T (OR146536 and OR146537)	N and T (OR146561)	N and T (OR146566, OR146567)
BCS17	N and T (OR146514)	N and T (OR146538)	N and T (OR146563)	N and G (OR146574)

Information referred to segments DNA-A, DNA-B, DNA-C and DNA-D. Abbreviatures: C (Cloning), S (Sanger sequencing), N (NGS), T (Trinity assembly) and G (Geneious), RP (RT-PCR). * (Incomplete nt and aa sequence)

### Diversity and phylogenetic analyses

The pairwise identities between nt and aa sequences and the overall mean distance were estimated. Pairwise identities were generally high for each segment (Additional file 1). Minimum pairwise identities for DNA-A were 97.5% in aa sequence and 75.7% in nt sequence. These identities were lower for DNA-B (82.5%) at aa level, but higher (80.2%) at nt level. DNA-C sequences showed higher values of identities of 87.6% at nt and at aa level, as well as DNA-D (92.2% in nt sequence and 93% in aa sequence). The average nucleotide diversity among DNA-A BcssDV1 sequences was 0.011 ± 0.001 among all sequences of Italy and Spanish samples. Mean diversities of all segments are shown in Table 4. Although mean diversities had low values, the highest value in the entire population was obtained in DNA-C, while the lowest was obtained in DNA-A.

**Table 4:** Overall mean diversity for each BcssDV1 segment

Segment	Overall mean diversity	# Viral sequences	Sequence length
DNA-A	0.011 ± 0.001	14	1346–1440
DNA-B	0.061 ± 0.000	15	1067–1068
DNA-C	0.075 ± 0.007	13	786
DNA-D	0.042 ± 0.004	9	1130–1146

Values are expressed in mean diversity of the total number of sequences of all regions ± error

Phylogenetic relationship of BcssDV1 variants were analysed at nt and aa level. Sequences belonging to specific isolates and not pools were discarded except IBC1, which was the reference obtained from amplification of viral DNA, cloning and sequences. Consequently, some phylogenetic trees did not contain representative sequences of all pools but included sequences from samples of all regions.

DNA-A showed a common group in both phylogenetic trees that included BcGYDV2 and BcGV1 (from New Zealand and China), and the Italian and Spanish BcssDV1 variants organized in several well supported groups with no structure based on the region of origin (Additional file 2: Figs. S1 and S2). For both phylogenetic trees Tomato Yellow Leaf Curl Virus 1 (TYLCV1) nt sequence (GenBank; OQ466372.1) and Rep protein sequence (GenBank; WFQ89762) were used as outgroups. DNA-B nt sequences were grouped in two groups supported with high bootstrap values. Group1 included variants of pools BCS10, BCS13, BCS15 and BCS17 (from Penedés and La Rioja) and Group2 contained variants of BCS8, BCS10, BCS11, BCS12 and BCS14 (from Ribera del Duero, La Rioja and Penedés) (Additional file 2: Figure S3 and S4). Clustering of group 1 was conserved in the aa phylogenetic tree. For both phylogenetic trees TYLCV1 nucleotide sequence (GenBank; OQ466372.1) and CP sequence (GenBank; WFQ89760) were used as outgroups. DNA-C nt and aa sequences showed two conserved groups: group 1 included Italian variants of BCI1 (Italy), and group 2 included Spanish variants from different regions (Additional file 2. Fig. S5). However, at aa level the Spanish variants group was less organized (Additional file 2. Fig. S6). Finally, DNA-D nucleotide phylogenetic trees showed two clusters. Cluster one included sequences of pools BCS8, BCS11, and BCS12 (from Ribera del Duero and Penedés) and cluster 2 included variants of pools BCI1 (from Italy), BCS10, BCS15 and BCS17 (from Penedés and La Rioja). (Additional file 2. Fig. S7). However, at aa level the Spanish variants group was less organized, and only group one was formed (Additional file 2. Fig. S8). In summary, there was not a clear and common clusterization among all segments based in the country (Italy or Spain) or in the region inside Spain (Ribera del Duero, Penedés or La Rioja).

### Detection of BcssDV1 in *B. cinerea* strains

**Fig. 4:**
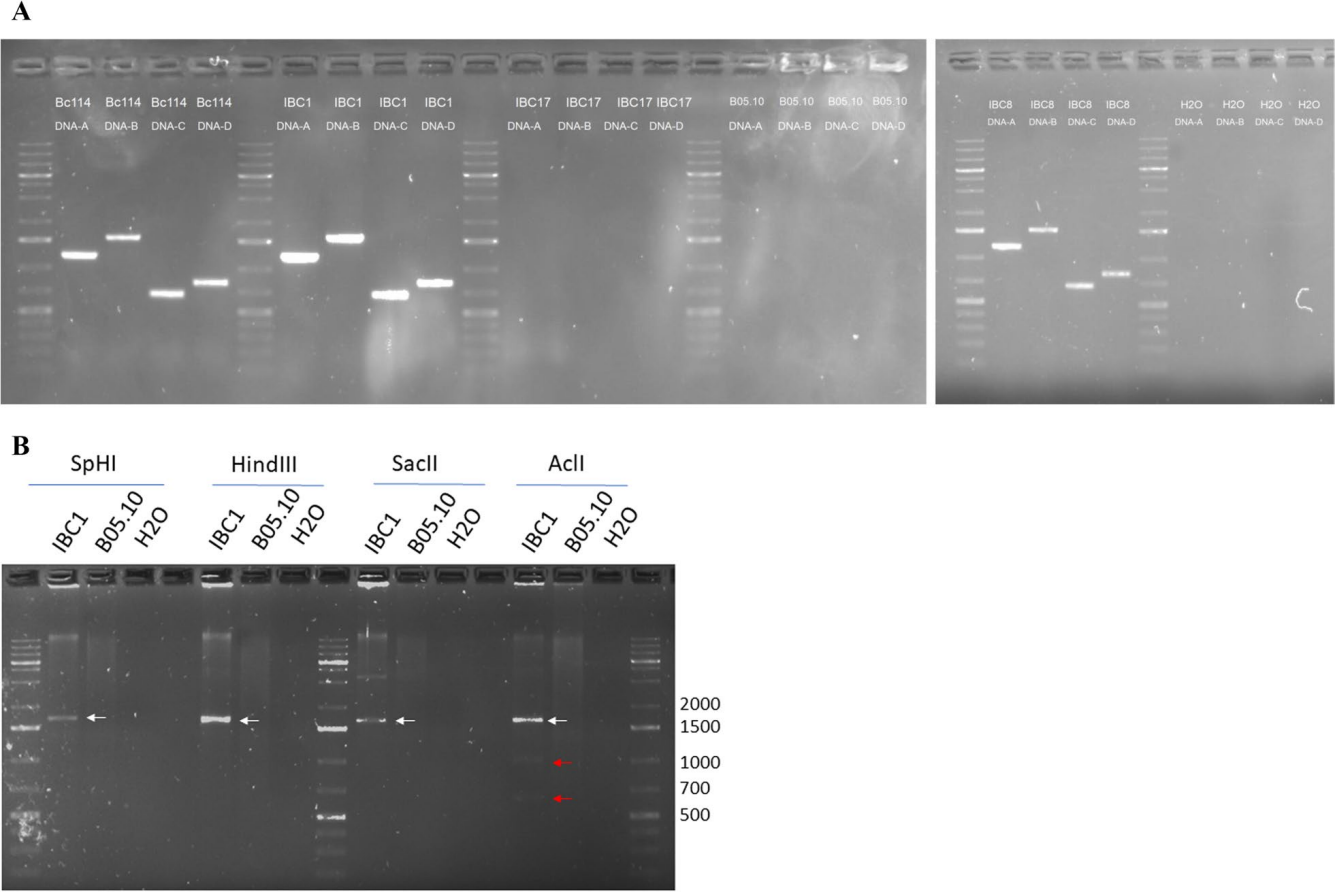
Detection of the four genomic segments of BcssDV1. Detection of the four genomic segments of BcssDV1. **A** Detection of the DNA-A, DNA-B, DNA-C and DNA-D belonging to BcssDV1 in infected field isolates. No detection of the virus in shown in non-infected fungal isolates BCI17 and B05.10, used as negative controls. **B** RCA and digestion of DNA-A, DNA-B, DNA-C and DNA-D. White arrows indicate linearized full-length genomic segments DNA-A, DNA-B, and DNA-C, and red arrows indicate the two fragments resulting from the double digestion of DNA-D. Several ladder bands size are indicated

BcssDV1 was detected in Spanish and Italian field isolates by RT-PCR using specific primers for DNA-A and DNA-B. From a total of 29 pools formed by 98 strains of Italy and 150 of Spain (a total of 248 isolates), BcssDV1 was detected by NGS in 10 pools. From these pools, composed by 84 field isolates, a first screening using primers for the detection of a partial sequence of DNA-B resulted in 18 infected isolates and 66 non-infected isolates or showing a very faint bands in the agarose gel. To confirm these results, additional RT-PCRs were carried out to detect DNA-A, which is generally more abundant than other segments. Out of these 66 isolates, 48 resulted positive, and 18 resulted negative. In summary, a total of 68 out of 85 isolates were infected by BcssDV1. Segments DNA-C and DNA-D were also detected in three analyzed field isolates Bc114, IBC8, IBC1 to show the co-occurrence of the four genomic segments when DNA-A was detected, there was not detection in the non-infected fungal isolates BCI17 from pool IBC1 and the control strain B05.10 (Fig. 4A). To demonstrate the presence of the four genomic segments, RCA products were digested with SphI, HindIII and SacII (NewEngland Biolabs) that have a single cut site on DNA-A, DNA-B, and DNA-C, respectively, generating linearized DNA sequences of ~ 1.7 kbp. Similarly, RCA product was digested with AclI (NewEngland Biolabs), that has a single cut in DNA-A, DNA-B, and DNA-C, and two cut sites in DNA-D, generating two linear DNA sequences of 1,1 kbp and 0,6 kpb, corresponding to the ~ 1.7 kbp of the DNA-D genomic segments (Fig. 4B).

Table 5 shows incidence values for all field isolates. All isolates of pool IBC1 (Lombardía, Italy) were infected with BcssDV1, and most pools showed incidence values over 75%. Pools BCS8 and BCS13, from Spanish vineyards of Ribera del Duero and La Rioja, showed an incidence of 22% and 50%, respectively. BcssDV1 is infecting at least eight isolates of each positive pool in Italy, and at least two isolates of each positive pool of Spain, with an overall incidence of 27.8% (69/248).

**Table 5:** Incidence of infection in isolates of pools of Spain and Italy

Pool	# positives	# pooled field isolates	Incidence (%)
BCI1	10	10	100
BCI2	8	9	89
BCS8	2	9	22
BCS10	7	7	100
BCS11	7	9	78
BCS12	6	7	86
BCS13	2	4	50
BCS14	11	12	92
BCS15	10	10	100
BCS17	6	8	75
TOTAL	69		

### Analysis of proteins structure

**Fig. 5:**
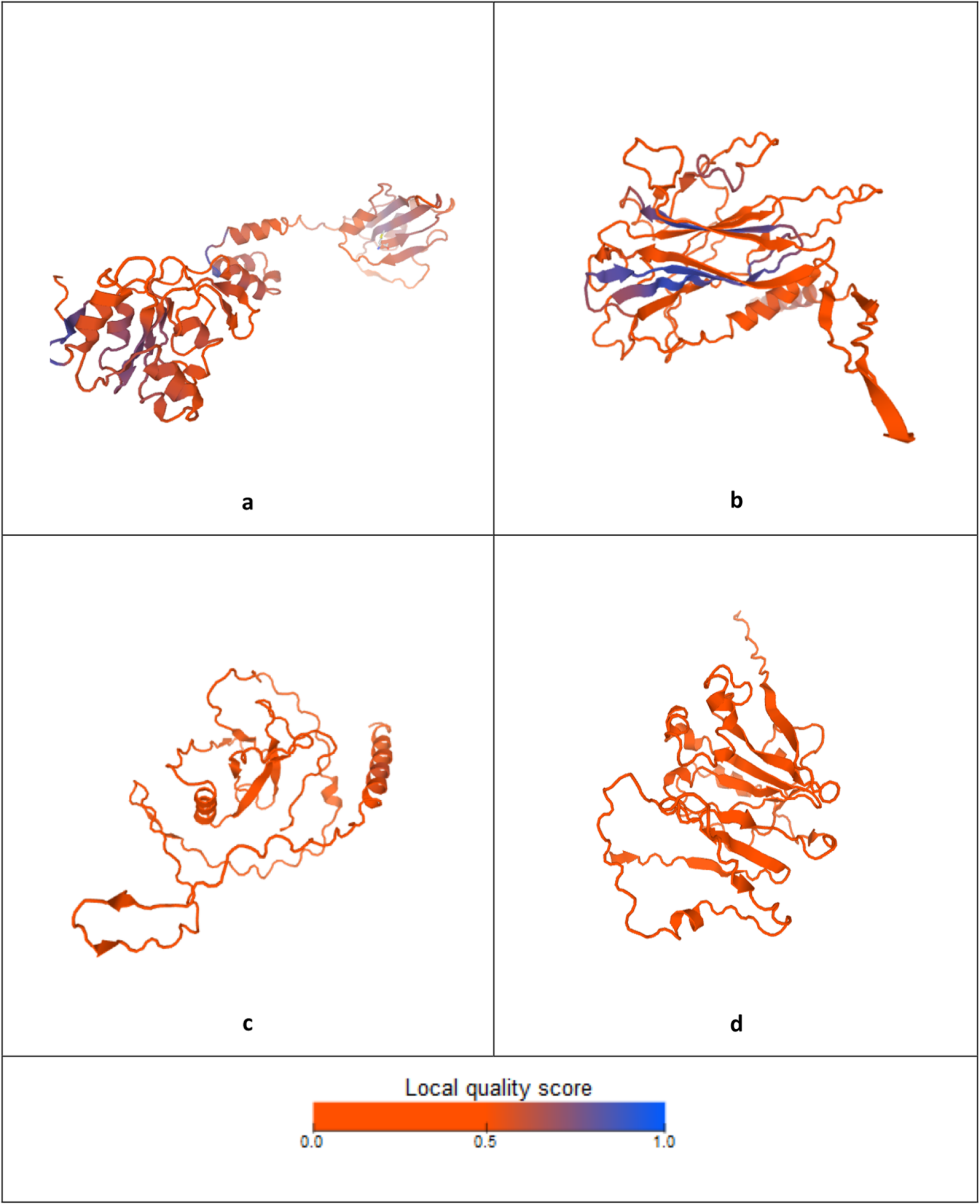
Model structures of BcssDV1 proteins coded by **a** DNA-A, **b** DNA-B, **c** DNA-C and **d** DNA-D Tridimensional structures and plots of QMEAN local quality estimates per protein. Blue tones indicate higher quality scores for each position in the predicted structure

**Fig. 6:**
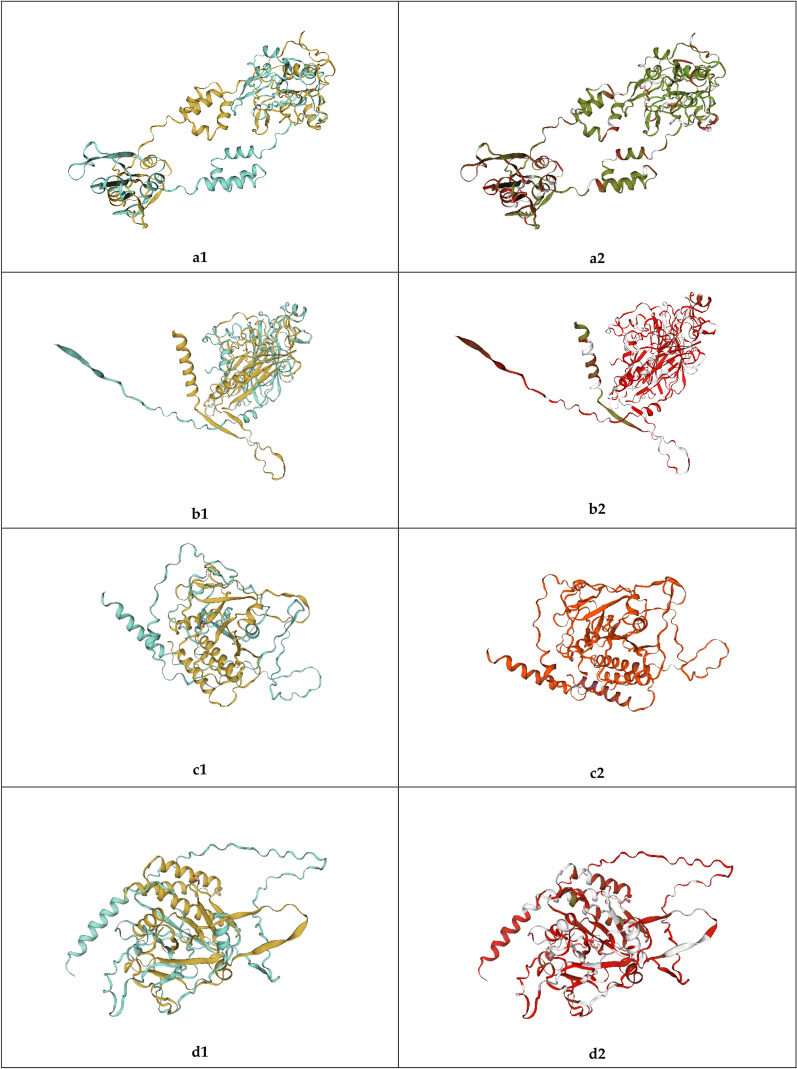
Superposition of predicted tertiary structures. Left column (1) showed superpositions of proteins with each chain marked in one colour (blue or yellow). Right column (2) showed same superpositions but indicating the degree of structural similarity of the alignment as a gradient from red to green that indicated the degree of structural similarity of the alignment. **a** Rep of BcssDV1 and FgGV1, **b** CP of BcssDV1 and FgGV1, **c** HP1 of BcssDV1 and hypothetical protein of FgGV1, **d** HP2 of BcssDV1 and hypothetical protein of FgGV1

NGS and posterior Blastx search have aided to identify new viral genomes and in the case of BcssDV1 to complete the molecular characterization of its genome composed by four genomic segments. Nevertheless, this information was not always able to elucidate the function of the proteins coded by each of genomic segments in the viral life cycle. To solve this limitation, prediction of the tertiary structures was performed with different software. AlphaFold2 predicted the complete 3-D structure of each of the proteins encoded by each genomic segment (Fig. 5, Additional file 3). Predicted structures of Rep and CP were compared and superposed with their corresponding proteins of FgGV1 and SsHADV1 (Fig. 6). Structural alignments showed some local regions of Rep and CP that presented structural homology in the superposition and were supported with high structural similarity. This value was based on the observed interatomic distances in the model with ensemble information extracted from structures that have been experimentally determined and are homologues to the target sequence [25]. Additionally, hypothetical proteins 1 (HP1) and 2 (HP2) predicted structures were compared with FgGV1 hypothetical protein, but, in this case, there was not high consistency in the superposition of tridimensional models.

Phyre2 and DALI were used to analyse tertiary structures of the proteins encoded in each segment and complement the characterization of hypothetical proteins (Additional file 4). Prediction of Rep, coded by DNA-A, showed three conserved regions. First, from 2 to 99 aa position, with high homology with wheat dwarf virus 1 Rep (PDBe: 6q1m) with 26% of identity and a 99.9% of confidence and with the DNA-binding domain of the replication initiation protein from the geminivirus TYLCV (RBD-like, PDBe: 1l5i) with 28% identity and a 99.9% of confidence. This motif consists of an HUH endonuclease involved in rolling-circle replication. Similar result was found with DALI, where same hit at PDBe: 6q1m was found with 24% of identity and 12.5 of Z-score (structural homology punctuation). Secondly, from position 163 to 211, the structure of primase-helicase of *Staphylococcus aureus* (PDBe: 7om0) was also identified with a 17% of identity and a 98.8 of confidence (Additional file 3), and from position 123 to 314 the primase-helicase of papillomavirus E1 (PDBe: 2v9p) with 98.2% and 18 (data not shown). This analysis confirms the role of DNA-A Rep in the rolling circle replication of the virus. Additional possible homologies were found, using DALI, with Replicator Initiator Protein from porcine circovirus PCV2 (PDBe: 2hw0) and with the Rep protein nuclease domain from the faba bean necrotic yellows nanovirus (PDBe: 6h8o), both circular single-stranded DNA viruses; and with an ATP-dependent protease ATPase subunit HslU (PDBe: 6pxk). The same analysis performed with the spliced Rep of 380 aa confirmed its implication in the replication cycle of BcssDV1 (data not shown). Additionally, similar results were obtained in the analysis of Rep FgGV1 and Rep SsHADV-1 (data not shown). Nuclear localization signals (NLS) [26] have been found in Rep of 321 aa (GPWLKKKCGA, position 17, score 6.5) and in the spliced Rep of 380 aa (DIQAPKRRKHTDRF, position 39, score 5.5; QAPKRRKHTD, position 41, score 6; GPWLKKKCGA, position 76, score 6.5).

In the prediction of the tertiary structure of CP, coded by DNA-B segment, DALI server identified homology with CPs of Ageratum yellow vein virus (PDBe: 6f2s) and faba bean necrotic stunt virus (PDBe: 6s44) with 9 and 12% of identity, and 9.5 and 8.8 of Z-score, respectively, both circular single-stranded DNA viruses. Phyre2 results showed structure homology in part of its structure, 52% of identity and a 40.6% of confidence with a eukaryotic translation initiation factor 4-e binding protein (PDBe: 2mx4) involved in cap-dependent translation; and approximately 30% of confidence and 33% of identity with endo-alpha-sialideases (PDBe: 1v0e), previously described in bacteriophages. These capsule-degrading tailspikes also promotes cellular plasticity and tumour metastasis in vertebrates [27]. Prediction of the tertiary structure of TYLCV CP revealed that there was a specific structural domain with homology from residue 40 to 238 of subunit h of CP of Ageratum yellow vein virus (PDBe: 6f2s). This subunit was characterized by X-ray and played a major role in the polarity of the protein by making the equatorial interface part of the protein [28]. However, this specific conserved domain of CP was not present in BcssDV1 aa sequence. Additionally, the analysis of FgGV1 and SsHADV1 using DALI also confirmed its implication in mycoviral encapsidation (data not shown).

**Fig. 7:**
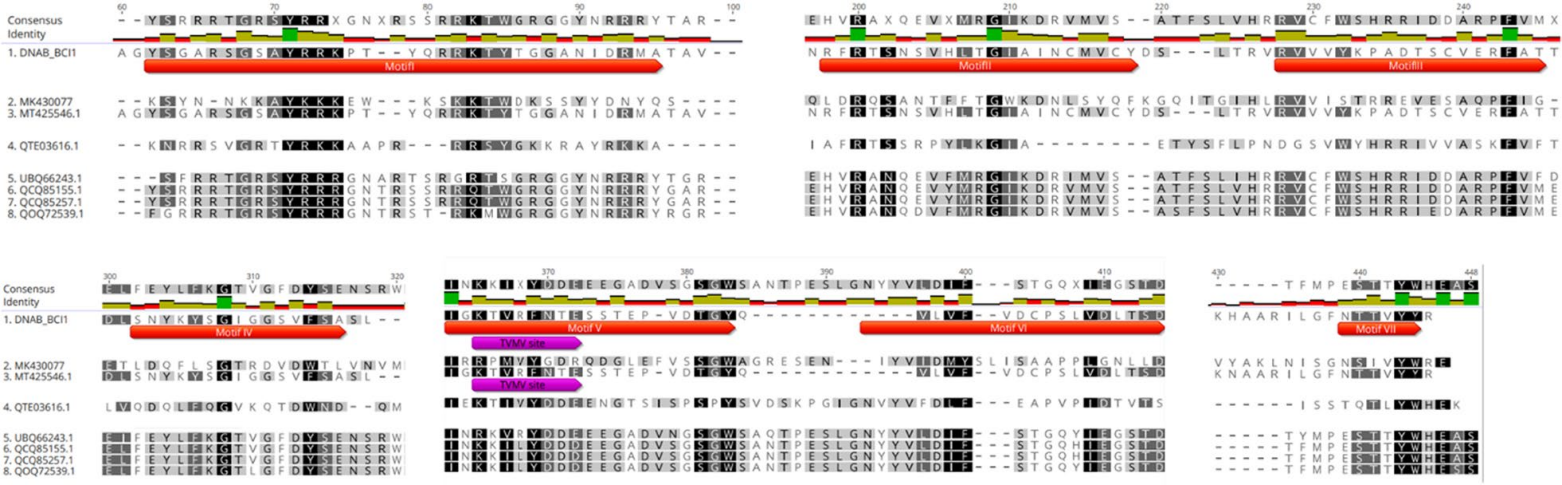
Alignment of amino acid sequences of capsid proteins of BcssDV1 and other genomovirus. Sequences of capsid proteins of BcssDV1, Fusarium graminearum gemytripvirus 1 (MK430077), Botrytis cinerea hypovirulence-associated DNA virus 1 isolate Skr4 (MT425546.1), Ficedula parva Genomoviridae sp. (QTE03616.1), Crane CRESS-DNA virus (UBQ66243.1), Finch associated genomovirus 7 (QCQ85155, QCQ85257.1) and bubaline-associated gemykrogvirus (QOQ72539.1). Red arrows indicate hypothetical conserved motifs of capsid proteins and purple arrows, predicted motifs with at least 50% of identity

In addition, a total of ten CP sequences of genomovirus that showed homology at least with 25% of identity with CP of FgGV1 were selected, aligned, and annotated with Geneious. As a result, seven potential conserved domains were detected through all sequence (Fig. 7). In putative Motif IV, a conservation with 50% of identity was found with tobacco vein mottling virus (TVMV) NIa protease recognition and cleavage site, essential for large polyprotein proteolytic processing in viruses of family *Potyviridae*.

DNA-C and DNA-D coded for two hypothetical proteins that did not share any conservation at nt or aa level with other viral proteins. After analysing their predicted tertiary structures, HP1 of DNA-C showed structural homology in some regions, previously described as predicted with high quality in the AlphaFold2 computational analysis. First hit showed structural homology with a 39% of identity and 59 of confidence level, from position 114 to 157, with typical domains of transferase, ephrin type-a receptor 2 of *Homo sapiens* (PDBe: 3c8x), involved in ephrin ligands regulate cell navigation during normal and oncogenic development [29]. Second hit showed structural homology with 81.8 of confidence and 37% of identity with ephrin receptor ligand binding domain of *Mus musculus* (PDBe: 1shw). Regarding DALI results, structural homology was found with Schlafen family member 11 (PDBe: 7zel) with 2.3 of Z-SCORE, which is an apoprotein containing two sequence domains, a putative DNA-binding domain and DNA/RNA helicase domain. This protein is a negative regulator of G1/S transition of mitotic cell cycle [30].

Structural homology of the hypothetical protein of DNA-D did not produce residues modelled at > 90% confidence. Phyre2 search predicted possible homologies with ctf19, kinetochore heterodimer of *Kluyveromyces lactis* (PDBe: 3zxu) implicated in cell cycle, with 71 of confidence and 43% of identity. DALI results also supported homology with a cell division cycle related protein kinase (CDC7) (6ya7) with 7% of identity and 4.2 of Z-SCORE. CDC7 activity is critical for the G1/S transition [31]. Phyre 2 determined, with lower level of confidence and percentage of identity, structural homology with RNA polymerase i-specific transcription initiation factor of *Saccharomyces cerevisiae* (PDBe: 5w65) was 62,4 and 18%, respectively.

Hypothetical protein of Fusarium graminearum gemyptripvirus 1 DNA-C (Genbank QIA59412) was also analyzed with Phyre2 to compare results with the ones obtained for BcssDV1 HPs, and the main result was an identity of 45% and confidence of 47 with the structure of the human 80 s ribosomal protein of *Drosophila melanogaster* (PDBe: 4v6w). Other hypothetical domains were Rps1 9E-like, a “winged helix” DNA-A binding domain of the ribosome of *Pyrococcus abyssi*, a hyperthermophile bacteria (PDBe: 2v7f) with 56,9 of confidence and 23% of identity and laminin subunit alpha-5 of *Homo sapiens* (PDB-e: 5xau), implicated in cell adhesion [32], with 52 of confidence and 13% of identity.

## Discussion

The full length genome of BcssDV1, a ssDNA virus belonging to family *Genomoviridae*, with a tetrasegmented genome, has been characterized in *B. cinerea* isolates of Italy and Spain. FgGV1 was the first described mycovirus with a multisegmented genome inside the family *Genomoviridae* [5]. Both FgGV1 and BcssDV1 possess a L-CR and a S-CR in all their genomic segments. Other ssDNA multisegmented viruses also exhibit these common regions in their segments, such as nanoviruses that contains CR-SL and CR-M or nanoviruses with CR-II [33]. The BcssDV1 S-CR also encompasses the proposed Ori, where the stem-loop structure displays similar secondary structures for all genomic segments. While other multisegmented geminiviruses as tomato golden mosaic virus (TGMV) or bean golden mosaic virus (BGMV) also share the same stem-loop structures [34], FgGV1 stem-loop structure differs in DNA-A and DNA-B with DNA-C.

BcssDV1 has been found in different regions of Italy and Spain with an overall incidence of 27.8%. From 98 samples of Italy and 150 of Spain, a total of 69 samples resulted positive by RT-PCR. Other mycoviruses infecting the fungus *B. cinerea* showed similar or lower incidences: Botrytis virus F showed 14.3% (12/84) in New Zealand and worldwide [35], Botrytis cinerea mitovirus 1 showed an overall incidence of 30% in different regions of Southern Spain (29/96) [36] and Botrytis cinerea mymonavirus 1 was only detected in four out of the 508 (0.8%) *B. cinerea* strains isolated from China [37]. However, there are examples of higher incidence rates in mycoviruses infecting other fungal hosts, such as Hymenoscyphus fraxineus mitovirus 1 (HfMV1), which displayed an incidence rate of 86% in the population of Sargans (N = 103 isolates; Eastern Switzerland) and 95% in the population of Aigle (N = 109 isolates; Western Switzerland) [38]. It is worth noting that BcssDV1 is not limited to Spain and Italy, as other variants have been identified in New Zealand (QLD98948.1) and China (QPB44148.1, UIX26077) [9, 10]. Given its high incidence and dispersal, it is plausible to suggest that BcssDV1 may also infect *B. cinerea* isolates worldwide in many different hosts.

Regarding mean pairwise genetic distances, DNA-C of BcssDV1 showed the highest mean pairwise identity (0.075 ± 0.007), while DNA-A displayed the lowest (0.011 ± 0.001). a partial sequence of Botrytis cinerea mitovirus 1 genome showed values of 0.023 ± 0.005 [36], higher than the mean value of pairwise identity found in DNA-A but lower than those obtained for segments DNA-B, DNA-C and DNA-D. In contrast, the mean pairwise identity of Botrytis virus F sequences (0.162 ± 0.021) was significantly higher than the ones obtained for each of the genomic segments of BcssDV1 [35]. The observed differences in incidence and nucleotide-level variability among these viruses can be attributed to their belonging to different viral families, possessing distinct types of genomes, and varying numbers of genomic segments.

Rep of other members of the family *Genomoviridae* display recognizable sequence similarity to Rep of member of the family *Geminiviridae*. In fact, DNA-A variants demonstrate high pairwise identities between aa sequences and contain characteristic aa Gemini_AL1 Rep catalytic conserved domains. In contrast, DNA-B, coding for the CP, and DNA-C, coding for the HP1, present the lowest minimum identities at aa level. This variability may be explained by the low values of sequence similarity previously found among CP and other hypothetical proteins of viruses belonging to the family *Genomoviridae* [6]. The obtained results suggest that the four BcssDV1 genomic segments do not followed the same evolution patterns. For instance, it has been observed that for bipartite begomoviruses, DNA-A and DNA-B respond differentially to evolutionary processes, with DNA-B being more permissive to variation [39]. Furthermore, evolutionary studies of FBNSV, formed by 8 segments, have demonstrated that each segment is subjected to variation and selection as an individual entity [34].

Phylogenetic analysis at nt or aa level reveals limited clusterization based on country or different and distant regions inside Spain, with Italian and Spanish samples clustering in different groups and Spanish isolates from La Rioja with tendency of forming groups with isolates from Penedés. Similar patterns of clusterization based on geographic regions have been observed in full-length genomes of other multisegmented ssDNA viruses like faba bean necrotic stunt nanovirus, which also formed two distinct groups, including different regions of Iran [40]. Additionally, begomoviruses have shown a clear geographic segregation of segments DNA-A and DNA-B, and some differences in the genetic structure of both [39].

FgGV1 was composed by three segments DNA-A, DNA-B and DNA-C, encoding Rep, CP, and a hypothetical protein, respectively. While DNA-A and DNA-B of FgGV1 were mutually interdependent and sufficient for their replication, DNA-C enhances the accumulation level of viral DNA in infected fungi and facilitates transmission via conidia [5]. Similar to FgGV1, DNA-A of BcssDV1, encoding Rep, contains the conserved domains of Gemini_AL1, while DNA-B encodes the hypothetical CP. BcssDV1 DNA-C and DNA-D both code for two hypothetical proteins without conserved motifs, which are presumed to be involved in different stages of the BcssDV1 life cycle. Tridimensional structure and function of the replication-associated protein has been deeply characterized in different viruses of family *Genomoviridae* [41–43].

The BcssDV1 Rep sequence not only contains Gemini_AL1 Rep catalytic conserved domains, but also exhibits structural homology with predicted tertiary conformation of Rep and DNA-binding domain of the replication initiation protein of members of the family *Geminiviridae*, the primase-helicase of a DNA virus of the family *Papillomaviridae* and an ATP-dependent protease ATPase. Geminiviruses encoded Rep plays an important role in the replication and transcription of the other viral genes and possesses DNA helicase and ATPase activity [44]. Geminiviruses replicate inside the nucleus, and Rep contains NLS, and removal of the aa of the NLS reduce nuclear import and nuclear accumulation of Rep [45]. BcssDV1 Rep (321 aa) also contains one NLS and spliced Rep (380 aa) contains three NLS, suggesting the importance of these NLS in the import and accumulation of the protein in the cell nucleus. Therefore, our results definitively confirm the role of BcssDV1 Rep in the life cycle of this mycovirus. Due to low conservation at sequence level of CP, HP1 and HP2 with other viral proteins, prediction of the tertiary structure of these proteins have allowed to propose their possible function in BcssDV1 life cycle. The BcssDV1 CP showed structural homology with CPs of ageratum yellow vein virus (AYVV) and faba bean necrotic stunt virus (FBNSV), other circular ssDNA viruses, confirming the function of DNA-B protein in the encapsidation of the mycovirus. AYVV is a plant geminivirus that form a two incomplete icosahedral particle, fused to form a geminate capsid [28]. FBNSV is a nanovirus whose distinct genome segments are encapsidated individually in icosahedral particles [46]. In the future, electron microscopy analysis will be conducted in order to analyse the structure of the BcssDV1 particles. Since the transcripts derived from BcssDV1 will be synthetized in the nucleus, it will be canonicals mRNA with 5’-CAP and 3’ poly-A. The first step in translation of 5′-capped host-cell mRNAs is the binding of the initiation factor eIF4F, that consists of three subunits. eIF4E binds to the cap, eIF4A is an RNA helicase and eIF4G connects the ribosome to the mRNA through eIF3 [47]. In plant potexviruses and carlaviruses CP interaction with this factor is necessary but not sufficient for facilitating the accumulation of these type of viruses [48]. BcssDV1 CP showed structure homology with a eukaryotic translation initiation factor 4-e binding protein, suggesting that could be involved in the interaction with eIF4E and possible in the accumulation of BcssDV1 inside its fungal host. The CP of geminiviruses contains NLS and is involved in the nucleocytoplasmic shuttling of the viral DNA [45], however BcssDV1 CP does not contain NLS. Notably, in this work, seven hypothetical conserved motifs were detected in CP sequences of viruses of the family *Genomoviridae* and other families, suggesting their potential importance in the initial stages of viral infection, could be through the interaction with a membrane cellular receptor, in viral particle assembly, in nucleocytoplasmic movement of proteins or DNA, or as a pathogenicity determinant as has been described for other geminiviruses [45]. Discovering new viruses of the family *Genomoviridae* and performing directed mutagenesis of these specific regions would help to elucidate their significance in viral structure and the role of the CP in interactions with the host. For BcsssDV1 DNA-C, HP1, structural homology was found with human ephrin type a receptor 2 transferases and signalling proteins of ephrin type-b, involved in regulation of cell–cell adhesion and motility in cancer cells [49]. In TYLCV1, the AC4 has a role in complementing the function of viral movement and degree of severity of the symptoms [45], then, HP1 of BcssDV1 may be contributing to movement and pathogenesis between the mycovirus and host cells. Interestingly, it was also found structural homology of DNA-C with an apoprotein with two sequence domains, a putative DNA-binding domain and DNA/RNA helicase domain. This protein is a negative regulator of G1/S transition of mitotic cell cycle. Then, since ssDNA mycovirus need to inhibit the DNA damage checkpoint, resulting in cell cycle progression while also stimulating DNA replication by preventing programmed cell death [45], BcssDV1 HP1 could be involved in the interaction with the DNA, maintaining separated the viral DNA duplex formed in the nucleus to facilitate the mycoviral replication cycle, instead to be related with the regulation of the cell cycle. Among all the analysed segments of BcssDV1, segment DNA-C was the most diverse among all variants and contains hypothetical motifs related to viral movement proteins. It is plausible that DNA-C represents the most susceptible segment within the viral genome for mutation and evolution, thereby facilitating future adaptation to yet undiscovered hosts. Considering this hypothesis, it is conceivable that HP1 shares functions with the movement protein of Bean golden mosaic virus (BGMV) and TGMV, both of which are considered essential factors in host adaptation, alongside other genes [50]. Furthermore, the description of new host of Sclerotinia sclerotiorum hypovirulence-associated DNA virus 1 (SsHADV-1) has been confirmed in *Penicillium olsonii* [51], raising the possibility of BcssDV1 infecting other ascomycetes.

In DNA-D HP2, structural homology was observed with the kinetochore heterodimer from yeast, which is involved in cell cycle, and with cell division cycle-related kinase. Results also supported homology with a cell division cycle related protein kinase (CDC7) which activity is critical for the G1/S transition of the replication cell cycle. Control and regulation replication cell cycle are some of the most important parts in the viral cycle. For instance, AC4 protein of TYLC1 acts as a viral “oncogene” stimulating cell DNA replication [45]. Additionally, HP2 also exhibited structural homology with RNA polymerase i-specific transcription initiation factor of *Saccharomyces cerevisiae* [52]. These factors play an essential role in cap-bearing cellular and viral RNAs, from the nucleus to the cytoplasm [49]. It could be that BcssDV1 HP2 acts stimulating replication cell cycle to favour BcssDV1 replication inside the nucleus. As another possible hypothesis, HP1 and HP2, which seem to have antagonistic effects on cell division cycle, could play a combined role in regulating it to favour replication of BcssDV1. Construction of a synthetic BcssDV1 would allow to determine the real function of their predicted proteins and study the mycoviral life cycle and its interaction with the fungal cell.

## Conclusions

Botrytis cinerea ssDNA virus, one of the first multisegmented virus described in the family *Genomoviridae*, infects *B. cinerea* isolates worldwide with overall high conservation of variants in all segments. It has been demonstrated that BcssDV1 variants are highly conserved among *B. cinerea* isolates worldwide. However, segment DNA-C has proven to be the most diverse segment, suggesting key role in future adaptation to other hosts. This limited genetic variance among BcssDV1 strains may offer significant advantages for the development of biocontrol strategies, leveraging its hypovirulent properties. Consequently, the potential application of BcssDV1 as a biocontrol agent can be explored across diverse geographic areas, similar to the successful utilization of SsHADV1 [12, 50].

### Supplementary Information

**Additional file 1. **Matrix of identities (%) between nucleotide and amino acid sequences of segments DNA-A, DNA-B, DNA-C and DNA-D of BcssDV1.

**Additional file 2. **Phylogenetic trees of nucleotide and amino acid sequences of segments DNA-A. DNA-B, DNA-C and DNA-D.

**Additional file 3.** Model structures of BcssDV1 proteins coded by **a** DNA-A, **b** DNA-B, **c** DNA-C and **d** DNA-D and plots of QMEAN local quality estimates. Blue tones indicate higher quality scores for each position in the predicted structure.

**Additional file 4.** Results of structural homologies modelled by Phyre2 and DALI in proteins of BcssDV1 and FgGV1.

## Data Availability

RNAseq raw data where micoviral sequences were detected are included in BioProject PRJNA632510. All nucleotide and amino acid were submitted in Genbank with following accessions numbers: OR146507 to OR146516, OR146518 to OR146525, OR146527, OR146529, OR146531, OR146532, OR146534 to OR146547, OR146549 to OR146561, OR146563 to OR146570, OR146572 to OR146577, and OR158040.
