# Supplementary material for: Molecular characterization of a tetra segmented ssDNA virus infecting Botrytis cinerea worldwide
Source: Virol J. 2023 Dec 19;20:306. doi: 10.1186/s12985-023-02256-z (PMC10731770; doi:10.1186/s12985-023-02256-z)
Supplement: Supplementary file 2 — Additional file 2. Phylogenetic trees of nucleotide and amino acid sequences of segments DNA-A. DNA-B, DNA-C and DNA-D. [file 12985_2023_2256_MOESM2_ESM.pdf]

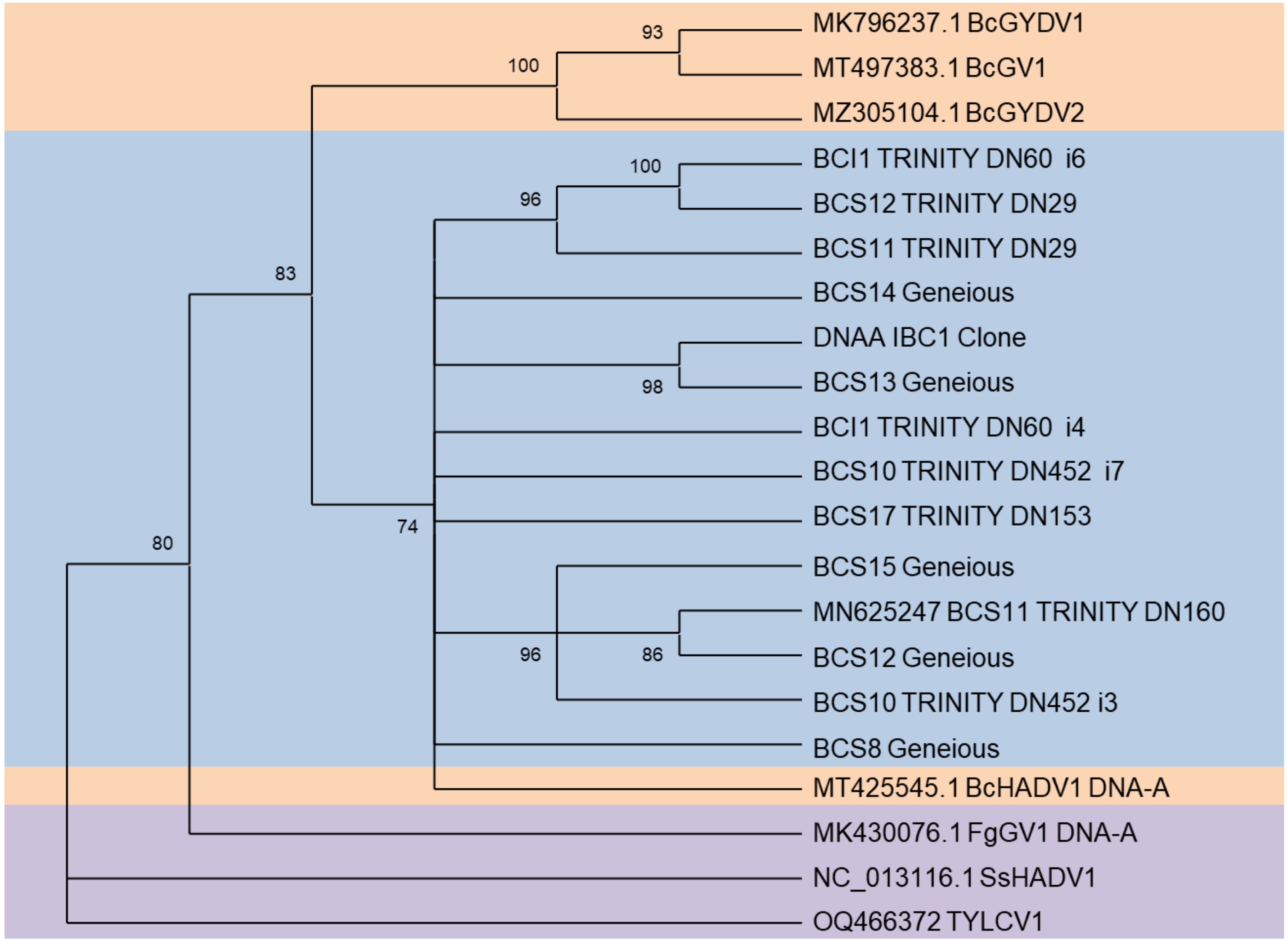

Figure 1. Phylogenetic tree based on nucleotide sequences of DNA-A. All bootstrap values (%) are represented at each node of the tree. A consensus tree was constructed from 1000 bootstrap trees (log likelihood of consensus tree: DNA-A, -12981.597)

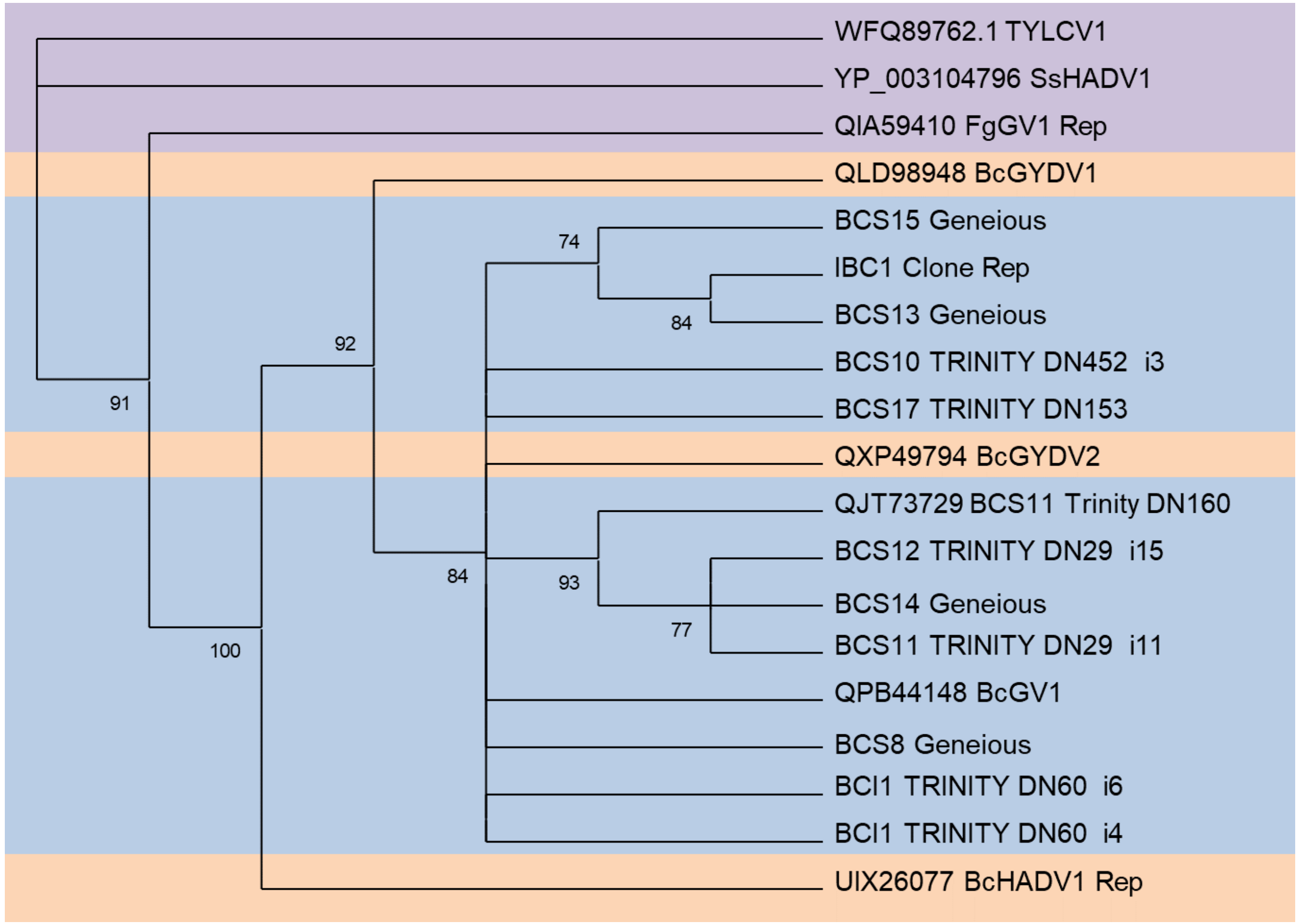

Figure 2. Phylogenetic tree based on amino acid sequences of DNA-A. All bootstrap values (%) are represented at each node of the tree. A consensus tree was constructed from 1000 bootstrap trees (log likelihood of consensus tree: DNA-A, -3592.4458)

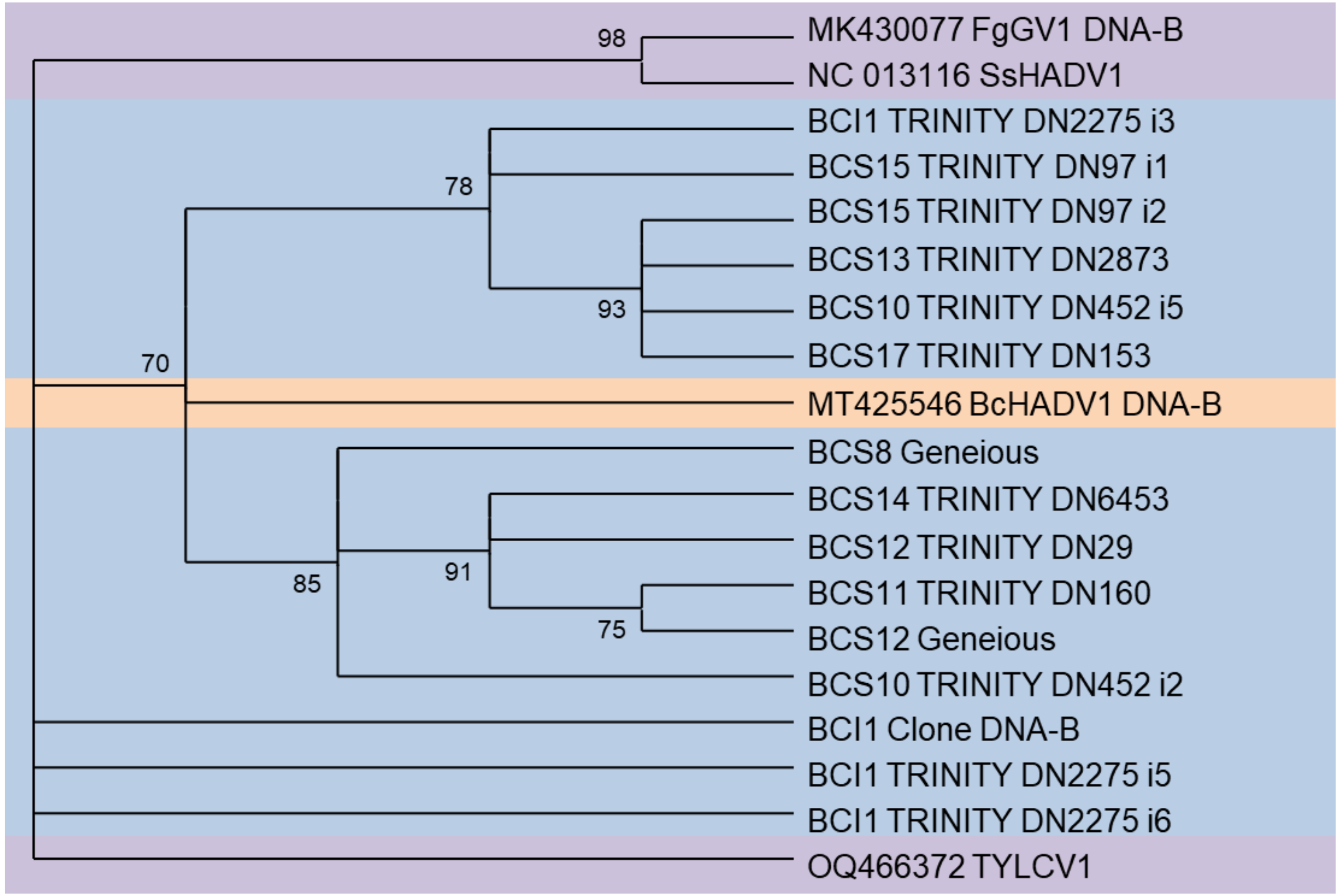

Figure 3. Phylogenetic tree based on nucleotide sequences of DNA-B. All bootstrap values (%) are represented at each node of the tree. A consensus tree was constructed from 1000 bootstrap trees (log likelihood of consensus tree: DNA-B, -6841.4621)

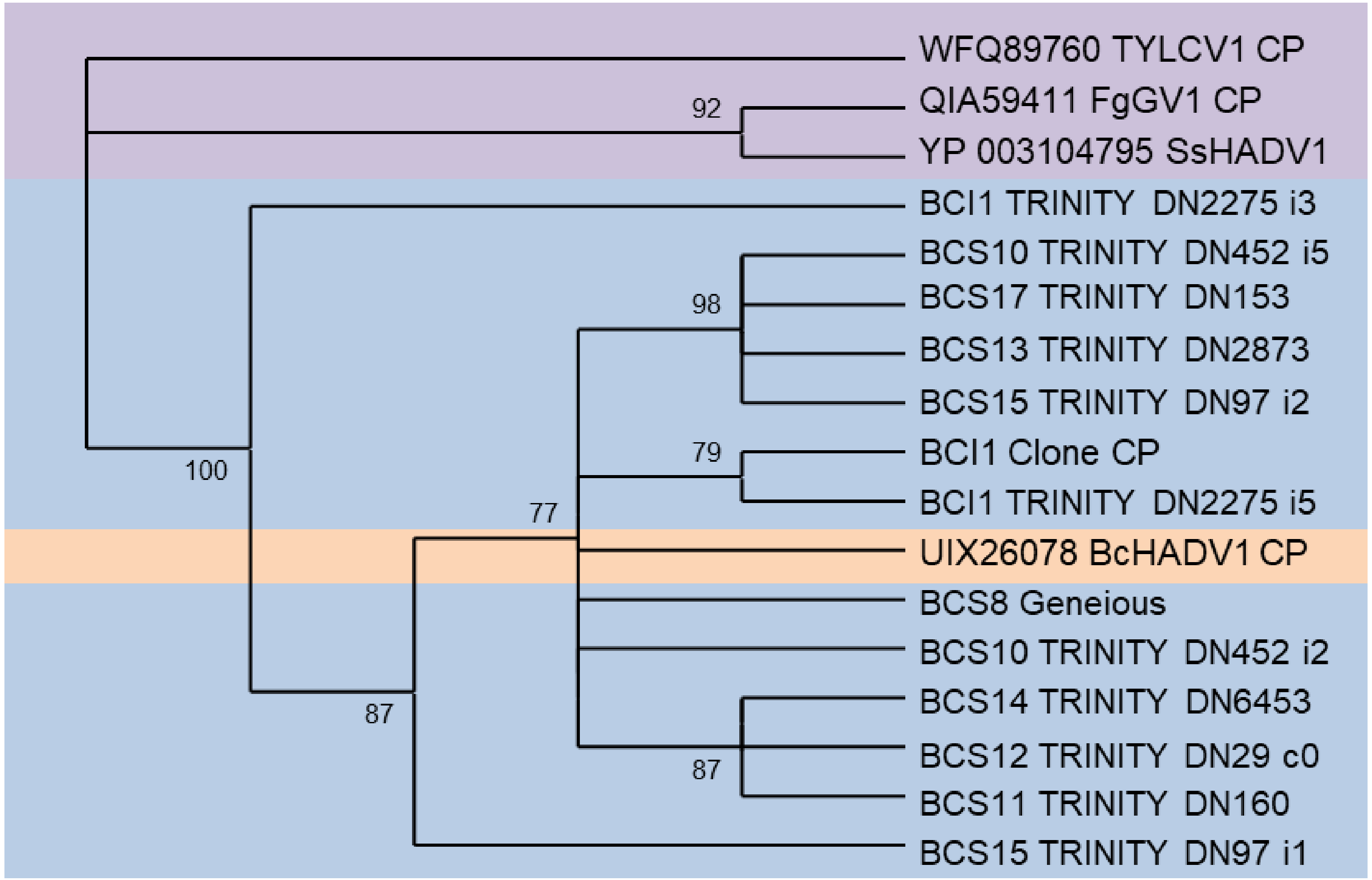

Figure 4. Phylogenetic tree based on amino acid sequences of DNA-B. All bootstrap values (%) are represented at each node of the tree. A consensus tree was constructed from 1000 bootstrap trees (log likelihood of consensus tree: DNA-B, -6841.4621)

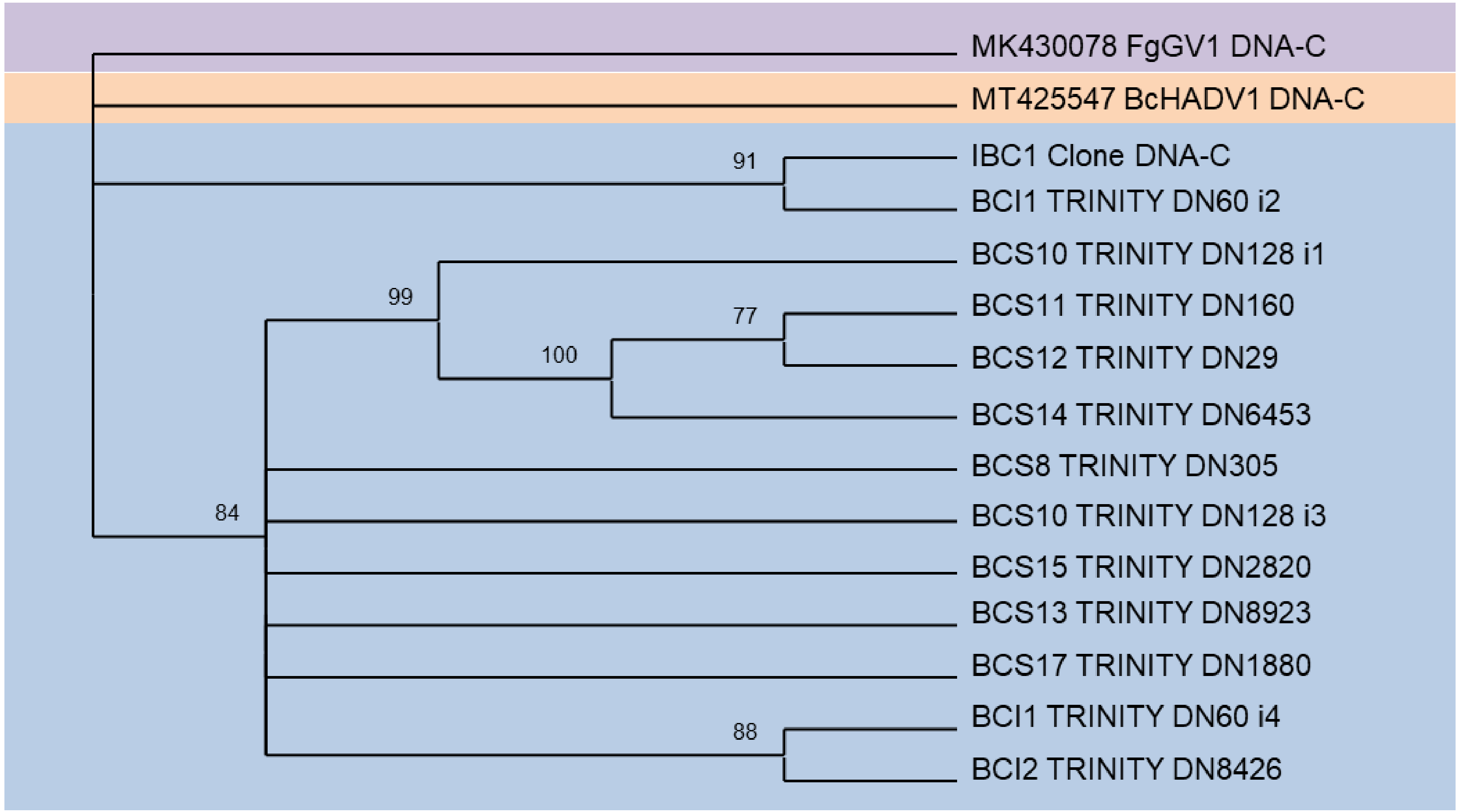

Figure 5. Phylogenetic tree based on nucleotide sequences of DNA-C. All bootstrap values (%) are represented at each node of the tree. A consensus tree was constructed from 1000 bootstrap trees (log likelihood of consensus tree: DNA-C, -3104.4721)

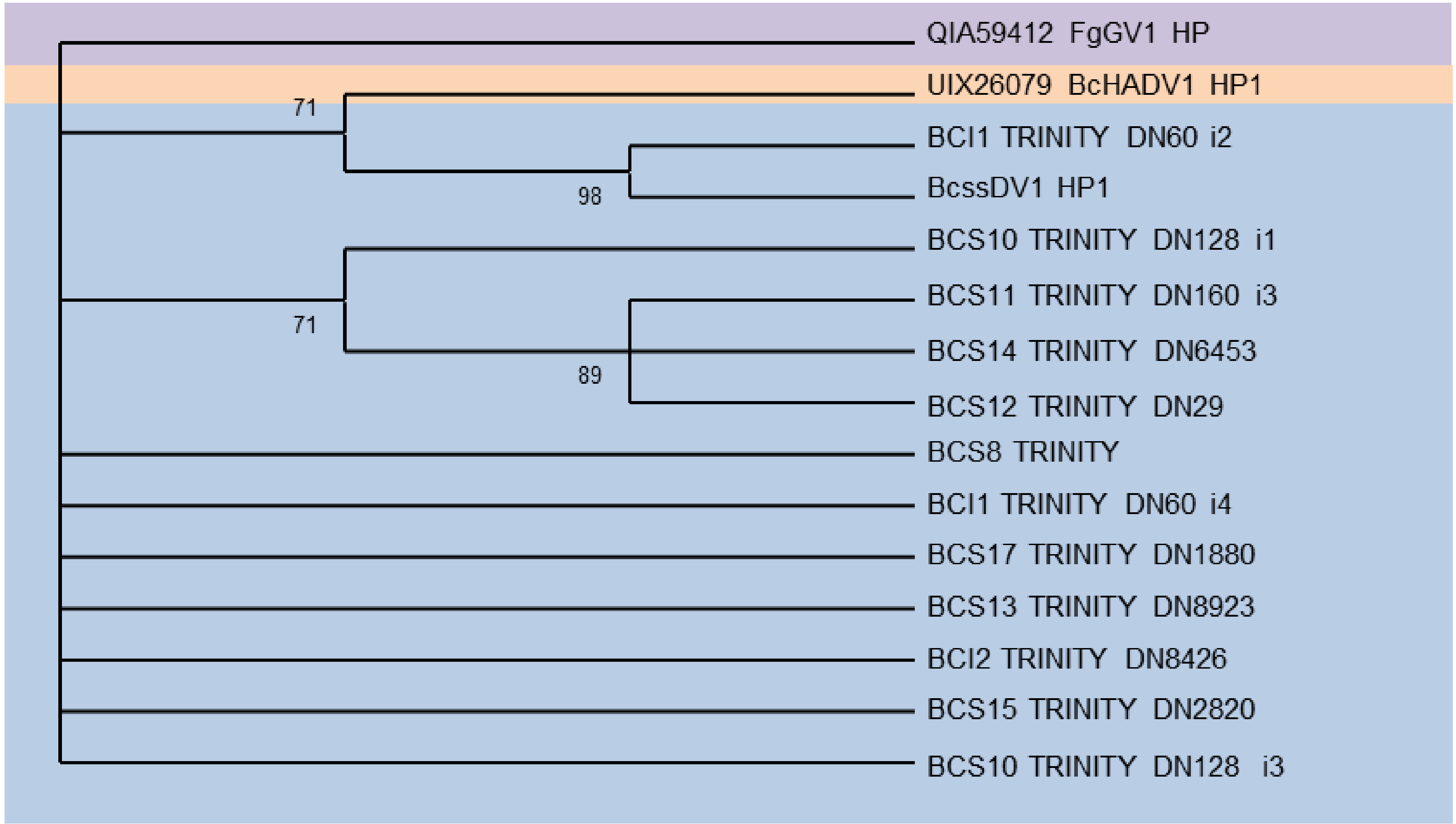

Figure 6. Phylogenetic tree based on amino acid sequences of DNA-C. All bootstrap values (%) are represented at each node of the tree. A consensus tree was constructed from 1000 bootstrap trees (log likelihood of consensus tree: DNA-C, -1692.1712)

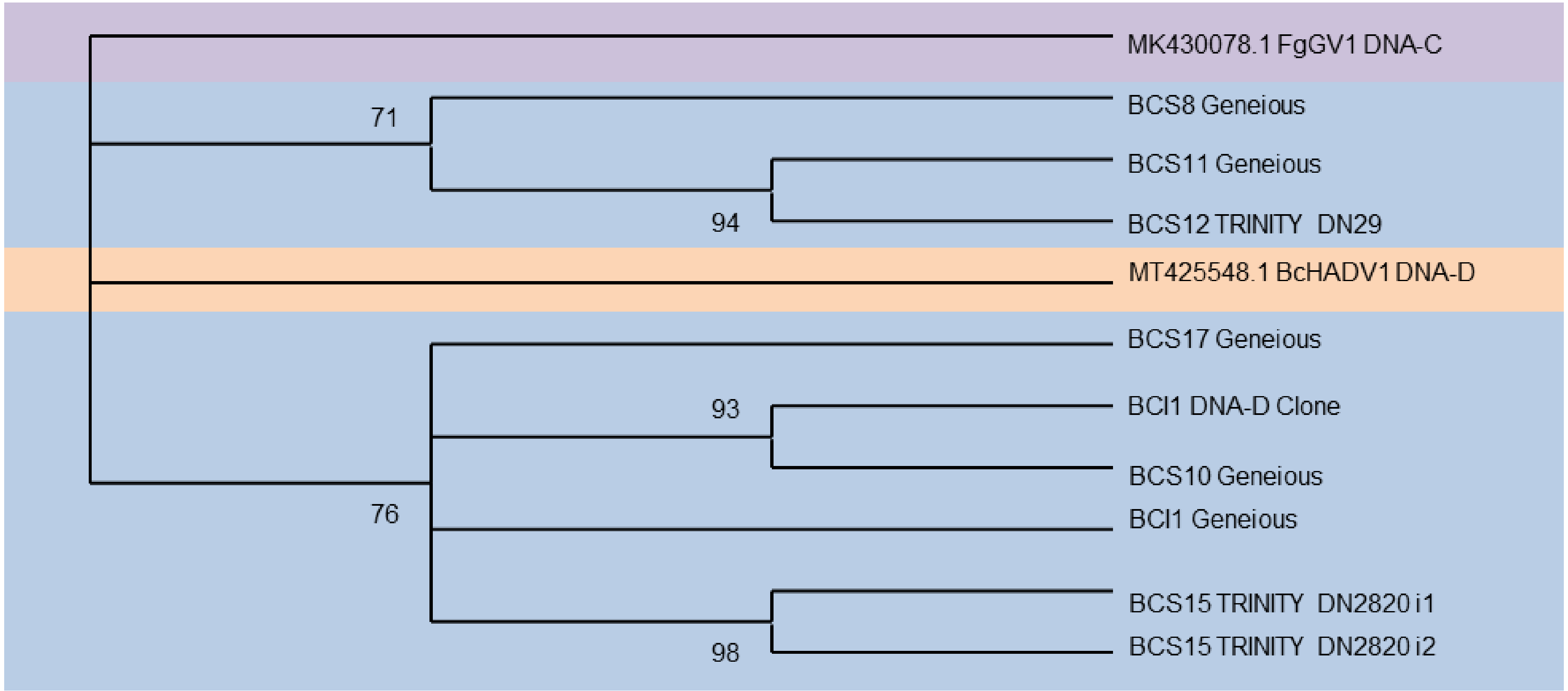

Figure 7. Phylogenetic tree based on nucleotide sequences of DNA-D. All bootstrap values (%) are represented at each node of the tree. A consensus tree was constructed from 1000 bootstrap trees (log likelihood of consensus tree: DNA-D, -3821.8583)

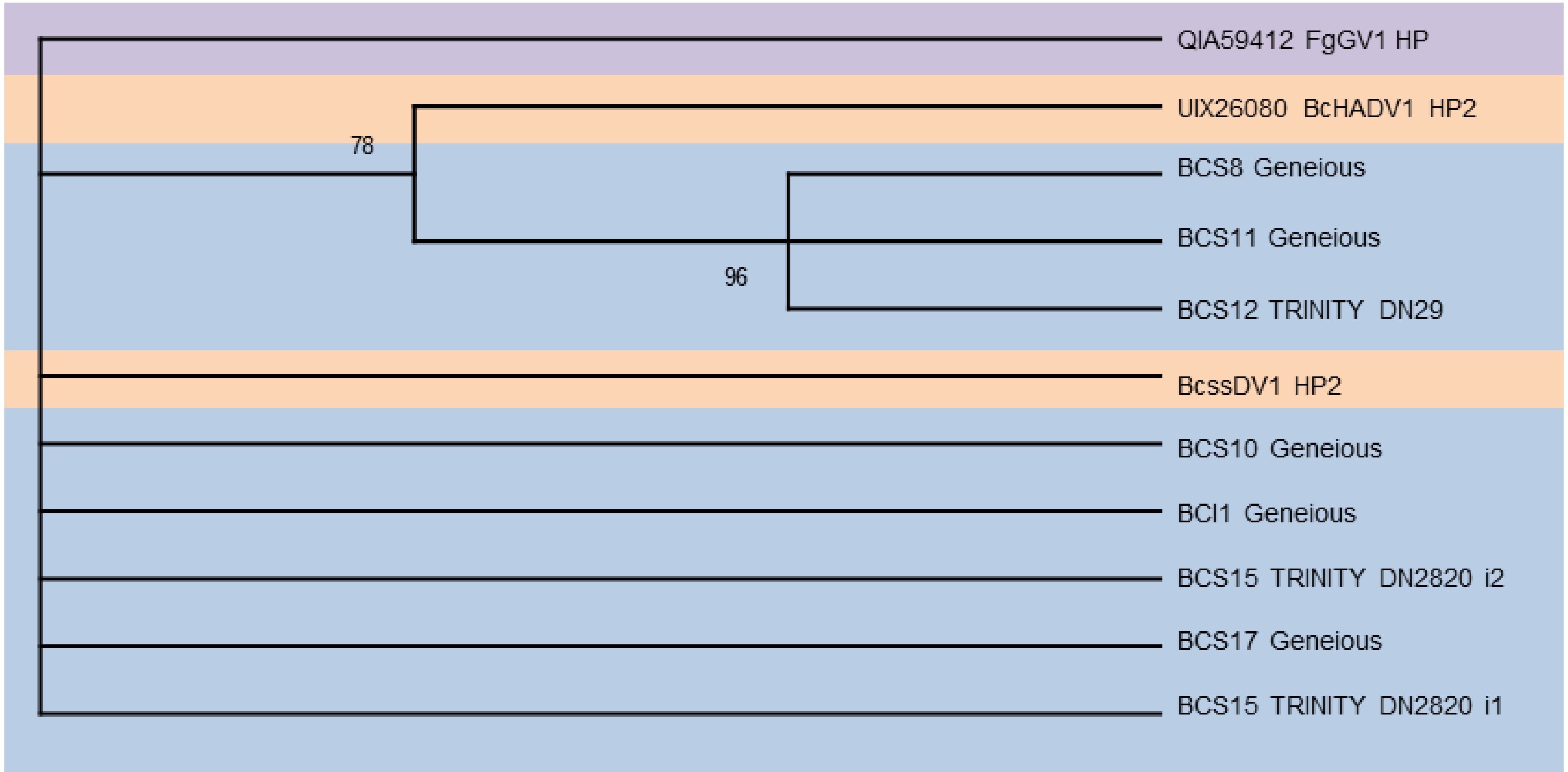

Figure 8. Phylogenetic tree based on amino acid sequences of DNA-D. All bootstrap values (%) are represented at each node of the tree. A consensus tree was constructed from 1000 bootstrap trees (log likelihood of consensus tree: DNA-D, -1652.9133)
