# Supplementary figures and images for: Molecular characterization of a tetra segmented ssDNA virus infecting Botrytis cinerea worldwide
Source: Virol J. 2023 Dec 19;20:306. doi: 10.1186/s12985-023-02256-z (PMC10731770; doi:10.1186/s12985-023-02256-z)

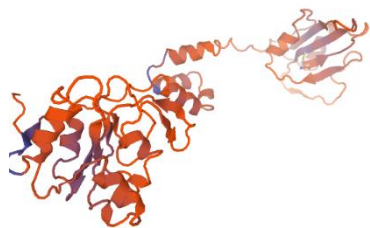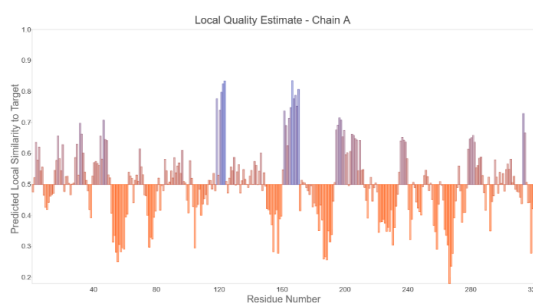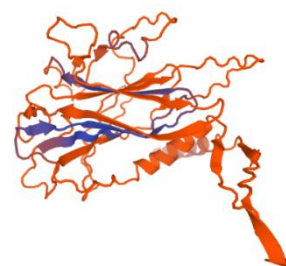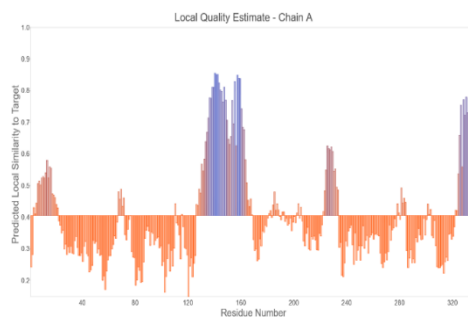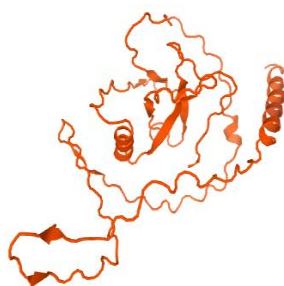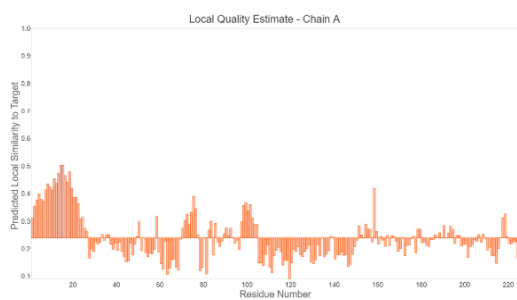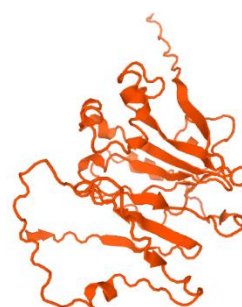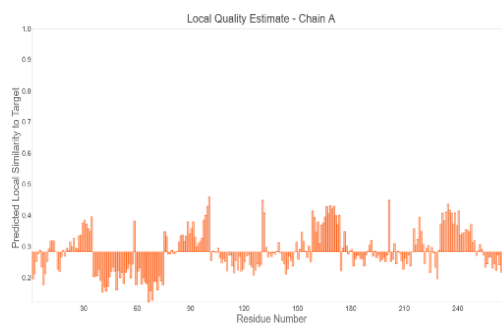

Local quality score

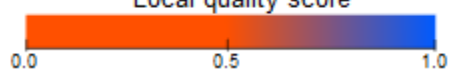

Supplement: Supplementary file 3 — Additional file 3. Model structures of BcssDV1 proteins coded by a DNA-A, b DNA-B, c DNA-C and d DNA-D and plots of QMEAN local quality estimates. Blue tones indicate higher quality scores for each position in the predicted structure. [file 12985_2023_2256_MOESM3_ESM.pdf]
