# Supplementary material for: Molecular characterization of a tetra segmented ssDNA virus infecting Botrytis cinerea worldwide
Source: Virol J. 2023 Dec 19;20:306. doi: 10.1186/s12985-023-02256-z (PMC10731770; doi:10.1186/s12985-023-02256-z)
Supplement: Supplementary file 4 — Additional file 4. Results of structural homologies modelled by Phyre2 and DALI in proteins of BcssDV1 and FgGV1. [file 12985_2023_2256_MOESM4_ESM.pdf]

| Sample | Phyre2 Results |
|---|---|

**DNA-A BcssDV1**

| # | Template | Alignment Coverage | 3D Model | Confidence | % i.d. | Template Information |
|---|---|---|---|---|---|---|
| 1 | c6q1mA | Alignment | 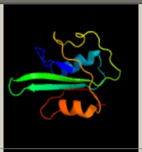 | 99.9 | 28 | **PDB header:**replication<br>**Chain:** A: **PDB Molecule:**replication-associated protein;<br>**PDBTitle:** crystal structure of the wheat dwarf virus rep domain<br>**PDB Entry:** PDBe RCSB PDBj |
| 2 | d1l5ia | Alignment | 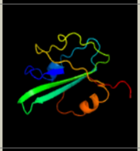 | 99.9 | 26 | **Fold:** Origin of replication-binding domain, RBD-like<br>**Superfamily:** Origin of replication-binding domain, RBD-like<br>**Family:** DNA-binding domain of REP protein<br>**PDB entry:** PDBe RCSB PDBj |
| 3 | c7om0C | Alignment | 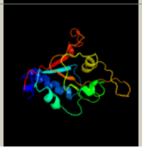 | 98.8 | 17 | **PDB header:**replication<br>**Chain:** C: **PDB Molecule:**dna primase;<br>**PDBTitle:** structure of primase-helicase in sapi5<br>**PDB Entry:** PDBe RCSB PDBj |

**DNA-B BcssDV1**

| # | Template | Alignment Coverage | 3D Model | Confidence | % i.d. | Template Information |
|---|---|---|---|---|---|---|
| 1 | c2mx4A | Alignment | 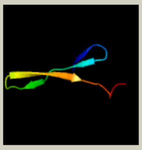 | 40.6 | 52 | **PDB header:**translation,protein binding<br>**Chain:** A: **PDB Molecule:**eukaryotic translation initiation factor 4e-binding protein<br>**PDBTitle:** nmr structure of phosphorylated 4e-bp2<br>**PDB Entry:** PDBe RCSB PDBj<br>Run Investigator |
| 2 | c1v0eB | Alignment | 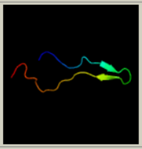 | 32.7 | 33 | **PDB header:**hydrolase<br>**Chain:** B: **PDB Molecule:**endo-alpha-sialidase;<br>**PDBTitle:** endosialidase of bacteriophage k1f<br>**PDB Entry:** PDBe RCSB PDBj<br>Run Investigator |
| 3 | d1v0ea1 | Alignment | 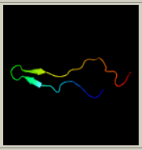 | 29.0 | 33 | **Fold:** 6-bladed beta-propeller<br>**Superfamily:** Sialidases<br>**Family:** Endo-alpha-sialidase<br>**PDB entry:** PDBe RCSB PDBj<br>Run Investigator |
| 4 | c6wxoB | Alignment | 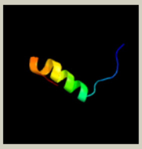 | 27.1 | 46 | **PDB header:**de novo protein<br>**Chain:** B: **PDB Molecule:**tfd-he;<br>**PDBTitle:** de novo tim barrel-ferredoxin fold fusion homodimer with 2-histidine2 2-glutamate centre tfd-he<br>**PDB Entry:** PDBe RCSB PDBj<br>Run Investigator |
| 5 | c4hceA | Alignment | 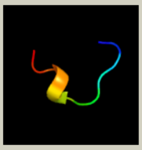 | 26.5 | 42 | **PDB header:**cell cycle<br>**Chain:** A: **PDB Molecule:**cell division control protein 13;<br>**PDBTitle:** crystal structure of the telomeric saccharomyces cerevisiae cdc13 ob22 domain<br>**PDB Entry:** PDBe RCSB PDBj<br>Run Investigator |
| 6 | c4hizA | Alignment | 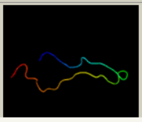 | 25.3 | 33 | **PDB header:**hydrolase,viral protein<br>**Chain:** A: **PDB Molecule:**endosialidase;<br>**PDBTitle:** phage phi92 endosialidase<br>**PDB Entry:** PDBe RCSB PDBj<br>Run Investigator |

**DNA-B FgGV1**

| # | Template | Alignment Coverage | 3D Model | Confidence | % i.d. | Template Information |
|---|---|---|---|---|---|---|
| 1 | d1ofda3 | Alignment | 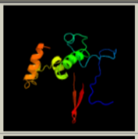 | 61.1 | 23 | **Fold:** Ntn hydrolase-like<br>**Superfamily:** N-terminal nucleophile aminohydrolases (Ntn hydrolases)<br>**Family:** Class II glutamine amidotransferases<br>**PDB entry:** PDBe RCSB PDBj<br>Phyre2 Run Investigator |
| 2 | d1ea0a3 | Alignment | 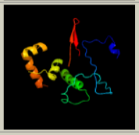 | 57.4 | 28 | **Fold:** Ntn hydrolase-like<br>**Superfamily:** N-terminal nucleophile aminohydrolases (Ntn hydrolases)<br>**Family:** Class II glutamine amidotransferases<br>**PDB entry:** PDBe RCSB PDBj<br>Phyre2 Run Investigator |
| 3 | c2lrqA_ | Alignment | 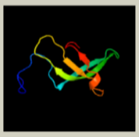 | 35.8 | 24 | **PDB header:**transcription<br>**Chain:** A: **PDB Molecule:**nua4 complex subunit eaf3 homolog;<br>**PDBTitle:** chemical shift assignment and solution structure of fr822a from2 drosophila melanogaster. northeast structural genomics consortium3 target fr822a<br>**PDB Entry:** PDBe RCSB PDBj<br>Phyre2 Run Investigator |
| 4 | d3byqa1 | Alignment | 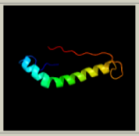 | 25.7 | 38 | **Fold:** Bacillus chorismate mutase-like<br>**Superfamily:** BB2672-like<br>**Family:** BB2672-like<br>**PDB entry:** PDBe RCSB PDBj<br>Phyre2 Run Investigator |
| 5 | c2cw1A_ | Alignment | 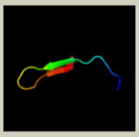 | 23.6 | 53 | **PDB header:**de novo protein<br>**Chain:** A: **PDB Molecule:**sn4m;<br>**PDBTitle:** solution structure of the de novo-designed lambda cro fold protein<br>**PDB Entry:** PDBe RCSB PDBj<br>Phyre2 Run Investigator |
| 6 | d2d0ta1 | Alignment | 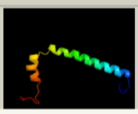 | 22.1 | 18 | **Fold:** Indolic compounds 2,3-dioxygenase-like<br>**Superfamily:** Indolic compounds 2,3-dioxygenase-like<br>**Family:** Indoleamine 2,3-dioxygenase-like<br>**PDB entry:** PDBe RCSB PDBj<br>Phyre2 Run Investigator |

**DNA-C BcssDV1**

| # | Template | Alignment Coverage | 3D Model | Confidence | % i.d. | Template Information |
|---|---|---|---|---|---|---|
| 1 | c3c8xA_ | Alignment | 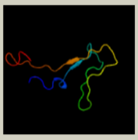 | 89.3 | 27 | **PDB header:**transferase<br>**Chain:** A: **PDB Molecule:**ephrin type-a receptor 2;<br>**PDBTitle:** crystal structure of the ligand binding domain of human ephrin a22 (epha2) receptor protein kinase<br>**PDB Entry:** PDBe RCSB PDBj<br>Phyre2 Run Investigator |
| 2 | d1shwb_ | Alignment | 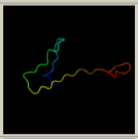 | 82.9 | 37 | **Fold:** Galactose-binding domain-like<br>**Superfamily:** Galactose-binding domain-like<br>**Family:** Ephrin receptor ligand binding domain<br>**PDB entry:** PDBe RCSB PDBj<br>Phyre2 Run Investigator |
| 3 | c3p1iC_ | Alignment | 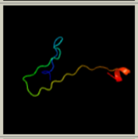 | 74.3 | 32 | **PDB header:**transferase,signaling protein<br>**Chain:** C: **PDB Molecule:**ephrin type-b receptor 3;<br>**PDBTitle:** ligand binding domain of human ephrin type-b receptor 3<br>**PDB Entry:** PDBe RCSB PDBj<br>Phyre2 Run Investigator |
| 4 | c2qbxB_ | Alignment | 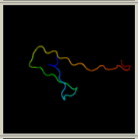 | 73.6 | 31 | **PDB header:**signaling protein<br>**Chain:** B: **PDB Molecule:**ephrin type-b receptor 2;<br>**PDBTitle:** ephb2/snew antagonistic peptide complex<br>**PDB Entry:** PDBe RCSB PDBj<br>Phyre2 Run Investigator |
| 5 | c7k7jA_ | Alignment | 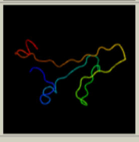 | 66.8 | 33 | **PDB header:**transferase<br>**Chain:** A: **PDB Molecule:**ephrin type-b receptor 6;<br>**PDBTitle:** ephb6 receptor ectodomain<br>**PDB Entry:** PDBe RCSB PDBj<br>Phyre2 Run Investigator |
| 6 | c3mbwA_ | Alignment | 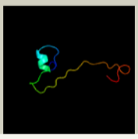 | 62.4 | 34 | **PDB header:**transferase/signaling protein<br>**Chain:** A: **PDB Molecule:**ephrin type-a receptor 2;<br>**PDBTitle:** crystal structure of the human ephrin a2 lbd and crd domains in2 complex with ephrin a1<br>**PDB Entry:** PDBe RCSB PDBj<br>Phyre2 Run Investigator |

DNA-C
FgGV1

| # | Template | Alignment Coverage | 3D Model | Confidence | % i.d. | Template Information |
|---|---|---|---|---|---|---|
| 1 | d2v7fa1 | Alignment | 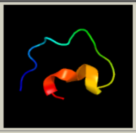 | 56.9 | 23 | **Fold:** DNA/RNA-binding 3-helical bundle **Superfamily:** "Winged helix" DNA-binding domain **Family:** Rps19E-like **PDB entry:** PDBe RCSB PDBj Run Investigator |
| 2 | c5xauD | Alignment | 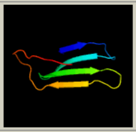 | 52.0 | 13 | **PDB header:**cell adhesion **Chain:** D: **PDB Molecule:**laminin subunit alpha-5; **PDBTitle:** crystal structure of integrin binding fragment of laminin-511 **PDB Entry:** PDBe RCSB PDBj Run Investigator |
| 3 | c5xyiT | Alignment | 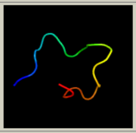 | 50.9 | 25 | **PDB header:**ribosome **Chain:** T: **PDB Molecule:**ribosomal protein s19e, putative; **PDBTitle:** small subunit of trichomonas vaginalis ribosome **PDB Entry:** PDBe RCSB PDBj Run Investigator |
| 4 | c3j38T | Alignment | 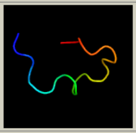 | 47.5 | 45 | **PDB header:**ribosome **Chain:** T: **PDB Molecule:**40s ribosomal protein s19a; **PDBTitle:** structure of the d. melanogaster 40s ribosomal proteins **PDB Entry:** PDBe RCSB PDBj Run Investigator |
| 5 | c3j3aT | Alignment | 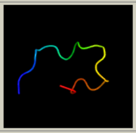 | 47.2 | 40 | **PDB header:**ribosome **Chain:** T: **PDB Molecule:**40s ribosomal protein s19; **PDBTitle:** structure of the human 40s ribosomal proteins **PDB Entry:** PDBe RCSB PDBj Run Investigator |
| 6 | c3zeyO | Alignment | 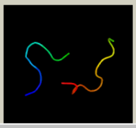 | 46.9 | 30 | **PDB header:**ribosome **Chain:** O: **PDB Molecule:**ribosomal protein s19, putative; **PDBTitle:** high-resolution cryo-electron microscopy structure of the trypanosoma2 brucei ribosome **PDB Entry:** PDBe RCSB PDBj Run Investigator |

DNA-D
BcssDV1

| # | Template | Alignment Coverage | 3D Model | Confidence | % i.d. | Template Information |
|---|---|---|---|---|---|---|
| 1 | c3zxuD | Alignment | 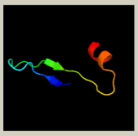 | 71.2 | 43 | **PDB header:**cell cycle **Chain:** D: **PDB Molecule:**ctf19; **PDBTitle:** crystal structure of the ctf19-mcm21 kinetochore heterodimer from2 yeast **PDB Entry:** PDBe RCSB PDBj Run Investigator |
| 2 | c5w65P | Alignment | 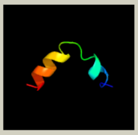 | 62.4 | 18 | **PDB header:**transcription **Chain:** P: **PDB Molecule:**rna polymerase i-specific transcription initiation factor **PDBTitle:** rna polymerase i initial transcribing complex state 2 **PDB Entry:** PDBe RCSB PDBj Run Investigator |
| 3 | c5n5xN | Alignment | 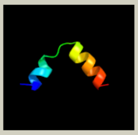 | 60.2 | 18 | **PDB header:**transcription **Chain:** N: **PDB Molecule:**rna polymerase i-specific transcription initiation factor **PDBTitle:** crystal structure of s. cerevisiae core factor at 3.2a resolution **PDB Entry:** PDBe RCSB PDBj Run Investigator |
| 4 | c6ohzA | Alignment | 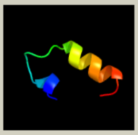 | 37.6 | 32 | **PDB header:**unknown function **Chain:** A: **PDB Molecule:**uncharacterized protein; **PDBTitle:** structure of an uncharacterized protein from leptospira borgpetersenii2 serovar hardjo-bovis (strain jb197) **PDB Entry:** PDBe RCSB PDBj Run Investigator |
| 5 | c3mi6A | Alignment | 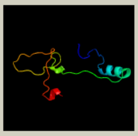 | 36.8 | 23 | **PDB header:**hydrolase **Chain:** A: **PDB Molecule:**alpha-galactosidase; **PDBTitle:** crystal structure of the alpha-galactosidase from lactobacillus2 brevis, northeast structural genomics consortium target lbr11. **PDB Entry:** PDBe RCSB PDBj Run Investigator |

Additional file 4a. Results of structural homologies modelled by Phyre 2 in proteins of BcssDV1 and FgGV1.

| Sample | DALI Results |
| --- | --- |
| DNA-A BcssDV1 | |

| No: | Chain | Z | rmsd | lali | nres | %id | PDB | Description |
| --- | --- | --- | --- | --- | --- | --- | --- | --- |
| 1: | 6q1m-A | 12.5 | 2.3 | 97 | 119 | 24 | PDB | MOLECULE: REPLICATION-ASSOCIATED PROTEIN; |
| 2: | 4gdf-A | 9.2 | 12.5 | 251 | 497 | 12 | PDB | MOLECULE: LARGE T ANTIGEN; |
| 3: | 3bos-B | 9.2 | 3.9 | 141 | 231 | 10 | PDB | MOLECULE: PUTATIVE DNA REPLICATION FACTOR; |
| 4: | 5zr1-A | 9.1 | 5.8 | 142 | 494 | 11 | PDB | MOLECULE: ORIGIN RECOGNITION COMPLEX SUBUNIT 1; |
| 5: | 2hw0-A | 9.1 | 3.6 | 96 | 115 | 18 | PDB | MOLECULE: REPLICASE; |
| 6: | 6pxk-K | 8.8 | 3.6 | 135 | 429 | 13 | PDB | MOLECULE: ATP-DEPENDENT PROTEASE ATPASE SUBUNIT HSLU; |
| 7: | 6h8o-A | 8.8 | 2.6 | 82 | 95 | 15 | PDB | MOLECULE: REPLICATION-ASSOCIATED PROTEIN; |
| 8: | 7jsf-A | 8.8 | 6.9 | 171 | 276 | 15 | PDB | MOLECULE: PROTEIN REP68; |
| 9: | 7tts-D | 8.7 | 6.3 | 145 | 463 | 12 | PDB | MOLECULE: CASEINOLYTIC PEPTIDASE B PROTEIN HOMOLOG; |
| 10: | 5v8f-D | 8.5 | 3.5 | 125 | 456 | 10 | PDB | MOLECULE: DNA REPLICATION LICENSING FACTOR MCM2; |
| 11: | 7jgr-D | 8.5 | 3.2 | 125 | 441 | 14 | PDB | MOLECULE: ORIGIN RECOGNITION COMPLEX SUBUNIT 2; |
| 12: | 1l8q-A | 8.4 | 3.4 | 126 | 321 | 14 | PDB | MOLECULE: CHROMOSOMAL REPLICATION INITIATOR PROTEIN DNAA; |
| 13: | 1ixs-B | 8.4 | 3.3 | 131 | 315 | 12 | PDB | MOLECULE: HOLLIDAY JUNCTION DNA HELICASE RUVA; |
| 14: | 7uqj-A | 8.3 | 3.9 | 138 | 585 | 12 | PDB | MOLECULE: ATPASE HISTONE CHAPERONE YTA7; |
| 15: | 5j1s-A | 8.3 | 3.5 | 133 | 277 | 15 | PDB | MOLECULE: TORSIN-1A; |
| 16: | 1g4a-E | 8.3 | 3.5 | 133 | 356 | 14 | PDB | MOLECULE: ATP-DEPENDENT HSL PROTEASE ATP-BINDING SUBUNIT |
| 17: | 3pvs-D | 8.1 | 3.1 | 122 | 424 | 11 | PDB | MOLECULE: REPLICATION-ASSOCIATED RECOMBINATION PROTEIN A; |
| 18: | 6iy8-A | 8.1 | 18.1 | 144 | 469 | 13 | PDB | MOLECULE: POSITIVE REGULATOR CAPR; |
| 19: | 6l1q-A | 8.1 | 3.9 | 138 | 267 | 14 | PDB | MOLECULE: CBBQ PROTEIN; |
| 20: | 7mi1-A | 8.0 | 6.5 | 151 | 2628 | 10 | PDB | MOLECULE: CHIMERA PROTEIN OF DYNEIN AND ENDOLYSIN; |
| 21: | 7mi8-A | 8.0 | 3.5 | 141 | 868 | 13 | PDB | MOLECULE: FUSION PROTEIN OF DYNEIN AND ENDOLYSIN; |
| 22: | 7jpp-E | 8.0 | 3.0 | 123 | 406 | 15 | PDB | MOLECULE: ORIGIN RECOGNITION COMPLEX SUBUNIT 1; |
| 23: | 5e7p-A | 7.9 | 6.0 | 134 | 719 | 10 | PDB | MOLECULE: CELL DIVISION CONTROL PROTEIN CDC48; |
| 24: | 7apd-B | 7.9 | 6.2 | 152 | 289 | 14 | PDB | MOLECULE: REPLICATION PROTEIN E1; |
| 25: | 7vcs-A | 7.8 | 10.1 | 154 | 766 | 10 | PDB | MOLECULE: TRANSITIONAL ENDOPLASMIC RETICULUM ATPASE; |
| 26: | 2qby-B | 7.8 | 3.1 | 130 | 368 | 17 | PDB | MOLECULE: CELL DIVISION CONTROL PROTEIN 6 HOMOLOG 1; |
| 27: | 7w42-B | 7.7 | 4.3 | 154 | 402 | 9 | PDB | MOLECULE: UNCHARACTERIZED ATPASE YJOB; |
| 28: | 8dr5-A | 7.7 | 7.2 | 136 | 646 | 12 | PDB | MOLECULE: REPLICATION FACTOR C SUBUNIT 1; |
| 29: | 7mi6-A | 7.6 | 12.0 | 167 | 2419 | 10 | PDB | MOLECULE: FUSION PROTEIN OF DYNEIN AND ENDOLYSIN; |
| 30: | 6z1f-3 | 7.6 | 4.2 | 134 | 284 | 13 | PDB | MOLECULE: RIBULOSE BISPHOSPHATE CARBOXYLASE/OXYGENASE ACTIV |
| 31: | 7qo4-M | 7.6 | 12.8 | 189 | 421 | 12 | PDB | MOLECULE: 26S PROTEASOME REGULATORY SUBUNIT RPN1; |
| 32: | 6mdm-D | 7.6 | 11.7 | 179 | 713 | 9 | PDB | MOLECULE: VESICLE-FUSING ATPASE; |
| 33: | 7tjj-I | 7.5 | 3.2 | 116 | 380 | 10 | PDB | MOLECULE: ORIGIN RECOGNITION COMPLEX SUBUNIT 1; |
| 34: | 6dgd-A | 7.5 | 4.6 | 126 | 704 | 10 | PDB | MOLECULE: PRIMOSOMAL PROTEIN N'; |
| 35: | 1w5s-B | 7.5 | 3.3 | 133 | 396 | 14 | PDB | MOLECULE: ORIGIN RECOGNITION COMPLEX SUBUNIT 2 ORC2; |

**DNA-B BcssDV1**

| No: | Chain | Z | rmsd | lali | nres | %id | PDB | Description |
|---|---|---|---|---|---|---|---|---|
| 1: | 6f2s-H | 9.5 | 3.3 | 152 | 218 | 9 | PDB | MOLECULE: CAPSID PROTEIN; |
| 2: | 6s44-A | 8.8 | 3.6 | 123 | 146 | 12 | PDB | MOLECULE: CAPSID PROTEIN; |
| 3: | 1r64-A | 8.7 | 10.4 | 132 | 481 | 4 | PDB | MOLECULE: KEXIN; |
| 4: | 4bcu-A | 8.4 | 4.1 | 149 | 188 | 11 | PDB | MOLECULE: COAT PROTEIN; |
| 5: | 6qfo-A | 8.2 | 5.7 | 113 | 1348 | 10 | PDB | MOLECULE: PEGA DOMAIN-CONTAINING PROTEIN,PEGA DOMAIN-CONTAI |
| 6: | 2wq4-A | 8.0 | 2.9 | 109 | 134 | 8 | PDB | MOLECULE: LECTIN; |
| 7: | 2xom-A | 8.0 | 2.7 | 104 | 145 | 5 | PDB | MOLECULE: ARABINOGALACTAN ENDO-1,4-BETA-GALACTOSIDASE; |
| 8: | 5azx-A | 7.9 | 2.8 | 92 | 100 | 8 | PDB | MOLECULE: TRANSMEMBRANE EMP24 DOMAIN-CONTAINING PROTEIN 10; |
| 9: | 3afg-B | 7.6 | 5.7 | 130 | 507 | 5 | PDB | MOLECULE: SUBTILISIN-LIKE SERINE PROTEASE; |
| 10: | 3wso-A | 7.6 | 3.1 | 122 | 252 | 8 | PDB | MOLECULE: F-BOX ONLY PROTEIN 44; |
| 11: | 2gsy-E | 7.4 | 3.4 | 121 | 433 | 5 | PDB | MOLECULE: POLYPROTEIN; |
| 12: | 5gu5-A | 7.3 | 2.8 | 93 | 108 | 9 | PDB | MOLECULE: TRANSMEMBRANE EMP24 DOMAIN-CONTAINING PROTEIN 5; |
| 13: | 4qxl-A | 7.2 | 3.5 | 103 | 115 | 15 | PDB | MOLECULE: FLAGELLAR PROTEIN FLHE; |
| 14: | 6yrf-A | 7.2 | 10.0 | 108 | 777 | 5 | PDB | MOLECULE: VEGETATIVE INSECTICIDAL PROTEIN; |
| 15: | 6vls-D | 7.1 | 11.6 | 106 | 963 | 7 | PDB | MOLECULE: MALTOSE/MALTODEXTRIN-BINDING PERIPLASMIC PROTEIN, |
| 16: | 4z11-A | 7.0 | 4.8 | 116 | 513 | 6 | PDB | MOLECULE: AURONE SYNTHASE; |
| 17: | 6wpx-B | 6.9 | 5.8 | 120 | 451 | 5 | PDB | MOLECULE: BLEST2; |
| 18: | 4gwm-A | 6.9 | 6.0 | 126 | 561 | 9 | PDB | MOLECULE: MEPRIN A SUBUNIT BETA; |
| 19: | 5gka-A | 6.9 | 3.4 | 123 | 253 | 7 | PDB | MOLECULE: CAPSID PROTEIN VP1; |
| 20: | 8e2f-A | 6.6 | 3.0 | 106 | 771 | 8 | PDB | MOLECULE: BACULOVIRAL IAP REPEAT-CONTAINING PROTEIN 6; |
| 21: | 4jdm-C | 6.6 | 3.1 | 108 | 269 | 10 | PDB | MOLECULE: VIRULENCE PLASMID PROTEIN PGP3-D; |
| 22: | 3iii-A | 6.5 | 7.6 | 129 | 556 | 7 | PDB | MOLECULE: COCE/NOND FAMILY HYDROLASE; |
| 23: | 6yf6-A | 6.5 | 6.6 | 108 | 140 | 8 | PDB | MOLECULE: GALACTOSE-BINDING LECTIN; |
| 24: | 2c26-A | 6.5 | 7.9 | 117 | 250 | 9 | PDB | MOLECULE: ENDOGLUCANASE; |
| 25: | 6e5c-A | 6.5 | 2.6 | 76 | 78 | 9 | PDB | MOLECULE: DE NOVO BETA PROTEIN; |
| 26: | 4k6b-B | 6.4 | 3.5 | 108 | 157 | 10 | PDB | MOLECULE: HS1 KNOB DOMAIN; |
| 27: | 6bqm-A | 6.4 | 14.0 | 107 | 460 | 11 | PDB | MOLECULE: SERINE PROTEASE VESC; |
| 28: | 5ta1-A | 6.4 | 11.4 | 135 | 627 | 5 | PDB | MOLECULE: GLYCOSIDE HYDROLASE; |
| 29: | 6za2-B | 6.4 | 11.1 | 115 | 1083 | 3 | PDB | MOLECULE: POR SECRETION SYSTEM PROTEIN PORU; |
| 30: | 1auy-B | 6.4 | 4.1 | 123 | 189 | 8 | PDB | MOLECULE: TURNIP YELLOW MOSAIC VIRUS; |
| 31: | 1stm-A | 6.4 | 3.2 | 112 | 141 | 7 | PDB | MOLECULE: SATELLITE PANICUM MOSAIC VIRUS; |
| 32: | 5yvl-D | 6.4 | 3.2 | 106 | 206 | 9 | PDB | MOLECULE: CYCLASE; |
| 33: | 7l2z-F | 6.4 | 3.2 | 114 | 664 | 9 | PDB | MOLECULE: CYCLIC DI-GMP-BINDING PROTEIN; |
| 34: | 6uwr-A | 6.4 | 13.5 | 117 | 660 | 6 | PDB | MOLECULE: ADP-RIBOSYLTRANSFERASE BINDING COMPONENT; |
| 35: | 3pvm-B | 6.3 | 4.7 | 126 | 1225 | 8 | PDB | MOLECULE: COMPLEMENT C5; |
| 36: | 4agg-A | 6.3 | 3.1 | 86 | 144 | 9 | PDB | MOLECULE: GALECTIN; |
| 37: | 8des-A | 6.3 | 4.7 | 150 | 475 | 8 | PDB | MOLECULE: MAJOR CAPSID PROTEIN; |
| 38: | 6tku-A | 6.3 | 4.0 | 116 | 545 | 8 | PDB | MOLECULE: DEPOLYMERASE KP32GP38; |
| 39: | 6mkb-B | 6.3 | 3.1 | 114 | 165 | 6 | PDB | MOLECULE: TUMOR NECROSIS FACTOR LIGAND SUPERFAMILY MEMBER 9 |
| 40: | 6i56-A | 6.3 | 11.9 | 104 | 279 | 6 | PDB | MOLECULE: PHAGE-LIKE ELEMENT PBSX PROTEIN XEPA; |

**DNA-C BcssDV1**

| No: | Chain | Z | rmsd | lali | nres | %id | PDB | Description |
|---|---|---|---|---|---|---|---|---|
| 1: | 7zel-A | 2.3 | 12.4 | 106 | 827 | 5 | PDB | MOLECULE: SCHLAFEN FAMILY MEMBER 11; |
| 2: | 3ajv-A | 2.3 | 3.3 | 73 | 168 | 12 | PDB | MOLECULE: PUTATIVE UNCHARACTERIZED PROTEIN; |
| 3: | 5yh1-A | 2.3 | 9.4 | 75 | 439 | 7 | PDB | MOLECULE: MEMBER OF S1P FAMILY OF RIBOSOMAL PROTEINS; |
| 4: | 7orl-A | 2.2 | 4.1 | 65 | 2183 | 8 | PDB | MOLECULE: RNA (5'-D(*(GTG))-R(P*AP*AP*UP*AP*CP*UP*AP*UP*AP* |
| 5: | 6qfj-A | 2.2 | 3.4 | 52 | 84 | 6 | PDB | MOLECULE: MAGNETOSOME MEMBRANE PROTEIN MAMB, PUTATIVE CO/ZN |
| 6: | 3fov-A | 2.2 | 3.4 | 60 | 102 | 5 | PDB | MOLECULE: UPF0102 PROTEIN RPA0323; |
| 7: | 5cet-A | 2.1 | 10.3 | 84 | 328 | 6 | PDB | MOLECULE: BIFUNCTIONAL OLIGORIBONUCLEASE AND PAP PHOSPHATAS |
| 8: | 1u6z-B | 2.1 | 7.2 | 86 | 500 | 5 | PDB | MOLECULE: EXOPOLYPHOSPHATASE; |
| 9: | 6gzb-A | 2.1 | 5.0 | 70 | 287 | 1 | PDB | MOLECULE: SPORE GERMINATION PROTEIN GERM; |
| 10: | 1n63-B | 2.1 | 4.1 | 63 | 805 | 2 | PDB | MOLECULE: CARBON MONOXIDE DEHYDROGENASE SMALL CHAIN; |

DNA-D
BcssDV1

| No: | Chain | Z | rmsd | lali | nres | %id | PDB | Description |
|---|---|---|---|---|---|---|---|---|
| 1: | 6ya7-A | 4.2 | 4.5 | 90 | 350 | 7 | PDB | MOLECULE: CELL DIVISION CYCLE 7-RELATED PROTEIN KINASE,CELL |
| 2: | 3u1w-C | 3.6 | 8.1 | 86 | 250 | 6 | PDB | MOLECULE: HYPOTHETICAL PERIPLASMIC PROTEIN; |
| 3: | 3w0s-A | 3.5 | 5.5 | 83 | 298 | 12 | PDB | MOLECULE: HYGROMYCIN-B 4-O-KINASE; |
| 4: | 7vnx-A | 3.5 | 4.4 | 89 | 216 | 11 | PDB | MOLECULE: TKARKI; |
| 5: | 6sum-A | 3.4 | 6.0 | 82 | 335 | 2 | PDB | MOLECULE: AMICOUMACIN KINASE; |
| 6: | 7s3l-A | 3.2 | 4.8 | 72 | 254 | 11 | PDB | MOLECULE: AMINOGLYCOSIDE PHOSPHOTRANSFERASE FAMILY PROTEIN; |
| 7: | 2xgl-A | 3.2 | 3.1 | 61 | 90 | 5 | PDB | MOLECULE: COLICIN-M IMMUNITY PROTEIN; |
| 8: | 6c9j-A | 3.2 | 4.5 | 82 | 386 | 12 | PDB | MOLECULE: 5'-AMP-ACTIVATED PROTEIN KINASE CATALYTIC SUBUNIT |
| 9: | 3i0o-A | 3.1 | 6.7 | 87 | 329 | 9 | PDB | MOLECULE: SPECTINOMYCIN PHOSPHOTRANSFERASE; |
| 10: | 5xzw-B | 3.1 | 6.8 | 101 | 379 | 6 | PDB | MOLECULE: SERINE/THREONINE-PROTEIN KINASE RAD53; |
| 11: | 3ats-A | 3.1 | 5.5 | 80 | 352 | 5 | PDB | MOLECULE: PUTATIVE UNCHARACTERIZED PROTEIN; |
| 12: | 2q83-A | 3.1 | 7.4 | 88 | 332 | 5 | PDB | MOLECULE: YTAA PROTEIN; |
| 13: | 5iqc-A | 3.1 | 6.5 | 88 | 301 | 5 | PDB | MOLECULE: BIFUNCTIONAL AAC/APH; |
| 14: | 4ocu-A | 3.0 | 5.8 | 90 | 356 | 8 | PDB | MOLECULE: N-ACETYLHEXOSAMINE 1-PHOSPHATE KINASE; |
| 15: | 8gwe-A | 3.0 | 4.7 | 87 | 931 | 8 | PDB | MOLECULE: RNA-DIRECTED RNA POLYMERASE; |
| 16: | 5dzt-A | 3.0 | 12.1 | 114 | 886 | 5 | PDB | MOLECULE: CYLM; |
| 17: | 2nyk-A | 3.0 | 8.2 | 90 | 237 | 7 | PDB | MOLECULE: M157; |
| 18: | 3pgb-A | 2.9 | 6.3 | 98 | 740 | 4 | PDB | MOLECULE: PUTATIVE UNCHARACTERIZED PROTEIN; |
| 19: | 7s9w-B | 2.9 | 14.4 | 107 | 529 | 3 | PDB | MOLECULE: DRMA; |
| 20: | 4hbr-A | 2.9 | 4.3 | 73 | 140 | 7 | PDB | MOLECULE: PUTATIVE PERIPLASMIC PROTEIN; |
| 21: | 5dzc-A | 2.9 | 17.5 | 95 | 816 | 4 | PDB | MOLECULE: CGMP-DEPENDENT PROTEIN KINASE, PUTATIVE; |
| 22: | 6rkw-B | 2.9 | 21.6 | 75 | 781 | 3 | PDB | MOLECULE: DNA GYRASE SUBUNIT A; |
| 23: | 3bb9-B | 2.9 | 2.9 | 58 | 125 | 7 | PDB | MOLECULE: PUTATIVE ORPHAN PROTEIN; |
| 24: | 4lv8-A | 2.9 | 4.8 | 91 | 360 | 13 | PDB | MOLECULE: RHOPTRY PROTEIN 5 C; |
| 25: | 3dxp-A | 2.8 | 5.3 | 79 | 329 | 9 | PDB | MOLECULE: PUTATIVE ACYL-COA DEHYDROGENASE; |
| 26: | 4gki-A | 2.8 | 5.6 | 76 | 272 | 1 | PDB | MOLECULE: AMINOGLYCOSIDE 3'-PHOSPHOTRANSFERASE APHA1-IAB; |
| 27: | 4ped-A | 2.8 | 6.2 | 100 | 387 | 8 | PDB | MOLECULE: CHAPERONE ACTIVITY OF BC1 COMPLEX-LIKE, MITOCHOND |
| 28: | 5ncl-A | 2.8 | 19.8 | 79 | 418 | 5 | PDB | MOLECULE: SERINE/THREONINE-PROTEIN KINASE CBK1; |
| 29: | 7wb4-f | 2.8 | 7.2 | 78 | 321 | 6 | PDB | MOLECULE: OUTER NUP133; |
| 30: | 5hl8-A | 2.8 | 2.8 | 49 | 81 | 8 | PDB | MOLECULE: TYPE II SECRETION SYSTEM PROTEIN L; |
| 31: | 6dzk-Y | 2.8 | 3.1 | 60 | 103 | 8 | PDB | MOLECULE: 16S RRNA; |
| 32: | 7zgr-A | 2.8 | 8.2 | 102 | 1253 | 3 | PDB | MOLECULE: PROTEIN CFT1; |

Additional file 4b. Results of structural homologies modelled by DALI in proteins of BcssDV1.
